# Immune Modulation in the Tumor Microenvironment: Bifurcation Analysis of Cancer-CTL-Monocyte Dynamics

**DOI:** 10.1007/s11538-025-01574-3

**Published:** 2025-12-24

**Authors:** Eymard Hernandez-Lopez, Russell Milne, Xiunan Wang

**Affiliations:** 1 Department of Mathematics, University of Tennessee at Chattanooga, 615 McCallie Ave, Chattanooga, TN 37403 USA; 2 Postgraduate and Research, TecNM-TESOEM, Paraje de San Isidro S/N, La Paz, 56400 México State México; 3 Department of Mathematical and Statistical Sciences, University of Alberta, Edmonton, Alberta T6G 2G1 Canada

**Keywords:** Quasi-steady-state approximation, Bistability, Allee effect, T cells, Monocyte-mediated immune activation

## Abstract

We present a mathematical model describing the interactions between cancer cells, cytotoxic T lymphocytes (CTLs), and monocytes within the tumor microenvironment. The model incorporates key immunological mechanisms, including tumor antigenicity, the Allee effect, and monocyte-mediated immune activation via MHCI cross-dressing. Using systems of nonlinear ordinary differential equations (ODEs), we derive analytical expressions for equilibrium points, evaluate their stability, and characterize bifurcations, such as saddle-node, Hopf, Bogdanov–Takens, and Bautin. A reduced model via quasi-steady-state approximation (QSSA) is also proposed, preserving the core dynamic structure to facilitate bifurcation analysis. A central finding of our study is the critical role of the monocyte-mediated T cell activation rate, denoted by the parameter $\beta$, which encapsulates the immunostimulatory potential of inflammatory monocytes presenting tumor antigens via MHCI cross-dressing. Numerical continuation corroborates the existence of multiple codimension-two organizing centers, delineating parameter regimes of tumor clearance, immune-mediated control, bistability, sustained oscillations, and inevitable escape. Our results quantitatively characterize the critical role of the monocyte-T-cell activation rate ($\beta$) and the Allee threshold ($\gamma$) in tipping the balance between immune surveillance and tumor persistence. This framework provides actionable bifurcation-based criteria for designing combination immunotherapies that enhance antigen presentation or monocyte functionality to shift the system toward tumor-eliminating attractors.

## Introduction

The complexity of the interaction between tumor cells and the immune system has driven the integration of experimental and mathematical approaches to understand and attempt to predict the dynamics of the antitumor response. In particular, the study Elewaut et al. (2024) suggests that cancer cells evade immunity by interfering with monocyte-mediated stimulation of T cells. This work highlights the importance of communication between cells within the tumor microenvironment and emphasizes how modulation of these processes could be helpful in the design of immunotherapies. Using mathematical models in this context makes it possible to incorporate and quantify the interactions between three key cellular populations: cancer cells, T lymphocytes, and monocytes. These models benefit from the conceptual clarity and quantitative rigor provided by differential equations, which facilitate the simulation of complex scenarios and the identification of critical thresholds in the immune response. Among these thresholds, the strong Allee effect plays a fundamental role.

The strong Allee effect is defined as the phenomenon by which the growth of a population becomes negative if its density or number of individuals falls below a critical threshold. In the case of tumor growth, this effect suggests that cancer cells must reach a minimum mass to sustain proliferation and activate cooperative mechanisms that promote immune evasion (Courchamp et al. 2008; Hernández-López and Wang 2025). Thus, including a term modeling a strong Allee effect in the dynamics of tumor cells not only realistically captures the need for a “critical niche,” but also allows for exploring therapeutic strategies to keep the tumor below that threshold. On the other hand, antigenicity in cancer refers to the ability of tumor cells to express antigens that the immune system can recognize. This characteristic is crucial for activating T lymphocytes and the consequent immune response against the tumor. The study highlights how the presence of cancer cells not only stimulates T cell responses through antigen presentation but paradoxically can also modulate this response by interfering with monocyte function (Tadepalli et al. 2023) and (Kwart et al. 2022). Incorporating a term representing antigenicity in mathematical models, for example, through a coefficient that amplifies T cell activation depending on tumor concentration, becomes essential for reproducing the behavior observed experimentally (Kirschner and Panetta 1998).

Combining these elements in a mathematical model makes replicating the interactions of the tumor and immune system comprehensively possible. Modeling through ordinary differential equations facilitates the interpretation of the underlying biological mechanisms and offers a tool to evaluate potential therapeutic strategies. Integrating both a strong Allee effect in tumor dynamics and an antigenicity term in T lymphocyte activation makes it possible to capture the complex feedback network that governs immune evasion, as demonstrated in Elewaut et al. (2024).

The article is organized as follows: In Section 2, the principal mathematical model is introduced, detailing its formulation, conceptual premises, and underlying dynamics; Section 3 presents a reduction of the original system to a simplified framework of two equations while preserving its essential properties. Subsequently, Section 4 addresses a bifurcation analysis of codimension 1, 2, and 3, identifying critical points and parametric configurations that govern qualitative changes in the system’s behavior. In Section 5, numerical continuation techniques are implemented to validate analytical results, ensuring consistency between theoretical predictions and computational simulations. Section 6 discusses the implications of the findings and explores potential applications in interdisciplinary contexts. Finally, Section 7 synthesizes the fundamental conclusions, emphasizing theoretical contributions, limitations, and future directions for research in nonlinear dynamics. This structure integrates analytical and numerical methodologies, providing a rigorous framework for studying complex systems involving interactions between cancerous cells and the immune system.

## Mathematical Model

We propose the following system of ordinary differential equations to describe the interaction between cancer cells, T lymphocytes, and monocytes within the tumor microenvironment, incorporating key biological mechanisms such as tumor growth subject to a strong Allee effect, immune activation driven by cancer antigenicity, and the immunostimulatory role of MHCI-dressed monocytes, as follows.

1 $$\begin{aligned} \frac{d C}{d t}= &  r C \left( 1 - b C\right) \left( C-\gamma \right) - \alpha C T,\nonumber \\ \frac{dT}{dt}= &  \lambda C +\beta M T - \delta T,\\ \frac{dM}{dt}= &  \rho - \eta M- \omega C M. \nonumber \end{aligned}$$

In this model *C*(*t*) denotes the density of cancer cells, *T*(*t*) the density of cytotoxic T lymphocytes (CTLs), and *M*(*t*) the density of functional monocytes capable of T cell activation. The cancer cell population follows a logistic-Allee dynamic, characterized by a growth rate *r*, a carrying capacity 1/*b*, and a critical population threshold $\gamma$ below which the tumor cannot sustain itself. Immune-mediated killing is represented by $\alpha C T$, reflecting T cell cytotoxicity.

The dynamics of T lymphocytes is driven by two key mechanisms. The first term, $\lambda C$, represents the direct activation of T cells through tumor antigenicity, where the presence of cancer cells improves the proliferation of T cells, likely mediated by antigen-presenting cells such as dendritic cells that process and present tumor-derived antigens. This captures the ability of the immune system to recognize and respond to tumor presence through antigen-dependent pathways. The second term, $\beta M T$, reflects the stimulation of T lymphocytes mediated by inflammatory monocytes. Recent evidence indicates that monocytes can acquire tumor antigens through MHCI cross-dressing, a process by which monocytes incorporate MHCI-tumor peptide complexes from tumor cells or dendritic cells and subsequently present these complexes to T cells, contributing to their activation and proliferation. This mechanism has been experimentally described in studies such as Elewaut et al. (2024), highlighting the relevance of MHCI-dressed monocytes in anti-tumor immunity. Variations in the parameter $\beta$ allow for indirect modeling of experimental conditions where this mechanism is active or suppressed, for example, in tumors lacking $\beta _{2}$-microglobulin ($\beta _{2}$m KO), where MHCI expression is impaired and consequently, cross-dressing-mediated antigen presentation is diminished.

Finally, the dynamics of the monocyte population include a recruitment rate $\rho$, a natural decay rate by the apoptosis mechanism $\eta M$, and a suppression term $\omega C M$ representing tumor-induced immunosuppressive effects on monocytes, either through apoptosis, functional exhaustion, or polarization into immunosuppressive phenotypes such as MDSC or M2-like macrophages, which lose the capacity to effectively stimulate T cells. For the sake of analytical tractability, particularly in stability analyses and threshold calculations, it is reasonable to set $\eta =0$. This simplification is justified under the assumption that the dominant mechanisms of monocyte depletion and inactivation within the tumor microenvironment are effectively captured by the $\omega C M$ term. In this way, processes such as apoptosis are considered implicitly within the tumor-driven suppression effect, allowing the model to retain biological plausibility while simplifying the mathematical expressions for further analysis.

Incorporating both $\lambda C$ and $\beta M T$ in the T cell dynamics is biologically justified, as they represent complementary immune activation pathways. The $\lambda C$ term captures direct tumor antigenicity-driven immune activation, while $\beta M T$ accounts for the indirect, monocyte-mediated stimulation enhanced by inflammatory monocytes presenting tumor antigens via MHCI cross-dressing. This dual mechanism reflects the complexity and redundancy of immune activation processes in the tumor microenvironment, providing greater biological realism and flexibility to the model. Moreover, the interaction term $\beta M T$ is particularly suitable as it captures the cell-cell interaction dependence, reflecting that T cell stimulation requires the simultaneous presence of both activated monocytes and T lymphocytes. This enables the model to account for immune synergy, where increasing densities of both populations enhance anti-tumor responses, a phenomenon frequently observed in immune-oncology studies.

In system (1) we do not include a direct tumor-killing term of the form $\kappa MC$. This modeling choice reflects the biological motivation of the study, where monocytes and monocyte-derived cells are assumed to contribute to anti-tumor responses primarily through antigen presentation and enhancement of cytotoxic T-cell activation, represented by the term $\beta M T$. Tumor-induced suppression of monocytes is already incorporated via the interaction term $\omega C M$, which captures the dominant effect described experimentally. To assess the robustness of this assumption, we additionally considered an extended model including the term $\kappa M C$. Numerical results show that, for biologically plausible values of $\kappa$, the qualitative structure is preserved; see Figure 13. Only unrealistically large killing rates significantly shift the thresholds.

### Critical Points

In this section, the critical points of the system will be analyzed and their stability will be examined. We first determine the equilibrium points of the system (1). To determine the equilibrium points, we solve the system of equations obtained by setting the right-hand sides of the differential equations to zero.

2 $$\begin{aligned} \begin{aligned} rC (1 - bC) (C - \gamma ) - \alpha C T&= 0, \\ \lambda C + \beta M T - \delta T&= 0, \\ \rho - \eta M - \omega C M&= 0. \end{aligned} \end{aligned}$$

Substituting $C = 0$ and solving for *T* and *M*, we obtain $T=0$ and $M=\rho /\eta$, which means the system admits a tumor–immune-free equilibrium $E_0$, given by:

3 $$\begin{aligned} E_0 = (0,0,\rho /\eta ). \end{aligned}$$

Once the point of the system has been identified, it is essential to analyze their stability to understand the local dynamics around each critical point. To achieve this, we compute the system’s Jacobian matrix and evaluate its eigenvalues at each equilibrium point. This analysis will allow us to determine the conditions under which the system exhibits behaviors such as tumor persistence, immune evasion, or tumor clearance. Next, we proceed with the local stability analysis by examining the eigenvalues of the Jacobian matrix.

### Local Stability Analysis

#### Proposition 2.1

Consider the tumor-free equilibrium $E_0 = (0,0,\rho /\eta )$. The stability of this equilibrium is as follows.

**(a)**: $E_0$ is locally asymptotically stable if $\beta \rho < \delta \eta$.
**(b)**: $E_0$ is a saddle if $\beta \rho > \delta \eta$.
**(c)**: $E_0$ is a degenerate equilibrium if $\beta \rho = \delta \eta$.

#### Proof

To analyze the stability of $E_0$, we compute the Jacobian matrix:

4 $$\begin{aligned} J = \begin{bmatrix} -r(\gamma + C(-2 + 3bC - 2b\gamma )) - \alpha T & -\alpha C & 0 \\ \lambda & \beta M - \delta & \beta T \\ -\omega M & 0 & -\eta - \omega C \end{bmatrix}. \end{aligned}$$

Evaluating *J* at $E_0 = (0,0,\rho /\eta )$, we obtain:

$$\begin{aligned} J(E_0) = \begin{bmatrix} -r\gamma & 0 & 0 \\ \lambda & \frac{\beta \rho }{\eta }-\delta & 0 \\ -\frac{\rho \omega }{\eta } & 0 & -\eta \end{bmatrix}. \end{aligned}$$

The eigenvalues of this matrix are:

$$\begin{aligned} \lambda _1 = -r\gamma , \quad \lambda _2 = -\eta , \quad \lambda _3 = -\delta + \frac{\beta \rho }{\eta }. \end{aligned}$$

For local asymptotic stability, all eigenvalues must have negative real parts. Clearly, $\lambda _1$ and $\lambda _2$ are negative. The sign of $\lambda _3$ determines the stability of $E_0$: If $\lambda _3 < 0$, then $E_0$ is locally asymptotically stable, which occurs when $\frac{\beta \rho }{\eta } < \delta$, or equivalently $\beta \rho < \delta \eta$. If $\lambda _3 > 0$, then $E_0$ has a positive eigenvalue, indicating a saddle point. If $\lambda _3 = 0$, then a bifurcation occurs, marking the transition between stability and instability.

Thus, the stability of $E_0$ is determined by the relationship between $\beta \rho$ and $\delta \eta$, as stated in the proposition. $\square$

When $C = 0$ in (2), the steady-state condition for the T-cell equation becomes

$$\begin{aligned} T(\beta M - \delta ) = 0, \end{aligned}$$

so that either $T = 0$ or $\beta M = \delta$. From the monocyte equation we obtain $M = \rho / \eta$, and therefore the nontrivial branch satisfies

$$\begin{aligned} M = \frac{\delta }{\beta } = \frac{\rho }{\eta } \quad \Longleftrightarrow \quad \beta \rho = \delta \eta . \end{aligned}$$

This condition coincides exactly with the degeneracy threshold identified in Proposition 2.1, which determines the change of stability of the tumor-free equilibrium $E_0 = (0,0,\rho /\eta )$. For this reason, the expression $M = \delta /\beta$ was not stated explicitly in the original version, although it is implicitly contained in the condition $\beta \rho = \delta \eta$ used in the stability analysis.

For coexisting states, we consider the system (1) and solve *M* from the third equation in terms of the variable *C*.

5 $$\begin{aligned} M=\frac{\rho }{\eta + \omega C }. \end{aligned}$$

Similarly, for the variable *T*, we solve the second equation of (1), which is initially expressed in terms of *M*. However, using the expression (5), we can rewrite it to obtain *T* in terms of *C*.

6 $$\begin{aligned} T=\frac{\lambda (\omega C + \eta )C}{\delta (\omega C+\eta )-\beta \rho }. \end{aligned}$$

Finally, we derive an expression solely in terms of *C*, which represents the critical points of the system (1) through the following equation.

7 $$\begin{aligned} \Gamma =\frac{(1-b C)(C-\gamma )\left( \delta (\omega C+\eta )-\beta \rho \right) }{C(\omega C +\eta )}, \end{aligned}$$

where $\Gamma =\alpha \lambda /r$. Since the expression for the coexistence equilibrium points (7) is algebraically complex, a direct analytical approach is impractical. Instead, a deeper investigation using bifurcation analysis is required to identify the thresholds at which the system’s stability changes. Through this approach, we can detect saddle-node bifurcations, which signal the emergence or disappearance of equilibrium points, as well as Hopf bifurcations, indicating transitions to periodic oscillations. In the following section, we will explore these phenomena through a detailed bifurcation analysis, providing a more comprehensive understanding of the system’s dynamics.

### Bifurcation in Model with Allee Effect

We now examine the system (1) that incorporates a strong Allee effect and an antigenicity term. This extended model exhibits a richer dynamical structure, including saddle-node, Hopf, and Takens-Bogdanov bifurcations. These bifurcations play a crucial role in determining the transitions between different regimes of tumor-immune interaction. The following results present a detailed characterization of these bifurcations, highlighting their impact on the stability and behavior of the system.

An alternative formulation of the expressions representing the critical points (7), obtained by extracting the main factor and the numerator, is as follows.

8 $$\begin{aligned} Q= r (1-b C) (C - \gamma ) (\delta (\eta + C\omega ) - \beta \rho ) -\alpha \lambda (\eta + C\omega )C, \end{aligned}$$

which will be used in the proofs of the subsequent results. This reformulation allows for a more structured analysis and facilitates the calculations of key properties.

#### Proposition 2.2

The set

9 $$\begin{aligned} S = \{ (b,r,\alpha ,\gamma ,\beta ,\rho ,\delta ,\eta , \omega ,\lambda ) \mid SN = 0 \} \end{aligned}$$

contains the saddle-node bifurcations of the system (1).

#### Proof

We consider the system (1) and compute the Jacobian matrix as given in equation (4). Next, we calculate the determinant of this matrix $\det (A)$. By factorizing the determinant $\det (A)$, considering its numerator, and using the expressions for the variables (5) and (6), we obtain the determinant solely in terms of $C$, as shown below.

$$\begin{aligned} detA_{C}= &  r (C ( 3 b C -2 (b\gamma + 1)) + \gamma ) (\beta \rho - \delta (\omega C + \eta ))^2 \\ &  + \alpha C\lambda \left( 2\delta (\omega C+ \eta )^2 - \beta \rho (3 \omega C + 2\eta ) \right) . \end{aligned}$$

Using the determinant of the Sylvester matrix as in Parker (1935), Woody (2016), Kuzovatov and Kytmanov (2025), Hernandez-Lopez et al. (2025), Hernandez-Lopez and Wang (2025) to find the common roots between polynomials (8) and $detA_{C}$, we derive the expression $SN$. $\square$

The expression for $SN$ is provided in Appendix A as (29). The analysis of the saddle-node bifurcation provided in the previous Proposition 2.2 establishes the conditions under which equilibrium points coalesce and disappear. However, in the dynamical behavior of the system (1), another fundamental bifurcation arises: the Hopf bifurcation, which governs the transition from equilibrium points to periodic oscillations.

To characterize this phenomenon, in the following theorem, we establish the necessary conditions under which a Hopf bifurcation occurs.

#### Theorem 2.3

The set

$$\begin{aligned} H = \{ (b,r,\alpha ,\gamma ,\beta ,\rho ,\delta ,\eta , \omega ,\lambda ) \mid \text {Hopf} = 0 \} \end{aligned}$$

contains the symmetric saddle and Hopf bifurcations of the system (1).

#### Proof

We consider the Jacobian matrix (4) of system (1) and compute its characteristic polynomial, which has the following form.

10 $$\begin{aligned} P({\psi })= \psi ^3 -a_{2} \psi ^2 +a_{1} \psi -a_{0}. \end{aligned}$$

In this case, the coefficients are given by $a_0$ as the determinant, $a_1$ as the sum of the second-order principal minors of matrix, and $a_2$ as the trace of (4). The conditions for obtaining one real root and two conjugate complex roots of the form $(a_{2} - \psi )(a_{1} + \psi ^2)$ are given by

$$\begin{aligned} a_0 - a_1 a_2 = 0. \end{aligned}$$

With this in mind, we can determine the sufficient conditions to find the bifurcation threshold as follows. Using the expressions (5) and (6), we express the coefficients $$ a_0, a_1, $$ and $$ a_2 $$ solely in terms of the density of cancer cells $$ C $$. Defining $$ H_0 = a_0 - a_1 a_2 $$, we find its common roots as in Parker (1935); Woody (2016); Kuzovatov and Kytmanov (2025), with the polynomial expression of the critical point (8), with respect to the variable $$ C $$. This results in the set that determines the Hopf bifurcations in terms of the system parameters. The entire expressions include (33) and (34). $$\square $$

The Hopf bifurcation analysis provides insight into the transition from steady-state to periodic oscillations in the interaction between cancer cells, effector $T$ cells, and monocytes $M$. However, in certain parameter regimes, a more complex bifurcation structure emerges, leading to a codimension-2 bifurcation known as the Bogdanov-Takens bifurcation. This phenomenon occurs when a saddle-node bifurcation and a Hopf bifurcation coincide, resulting in a degenerate equilibrium point with a double-zero eigenvalue. The Bogdanov-Takens bifurcation plays a crucial role in the global dynamics of the system, as it gives rise to homoclinic orbits, which can significantly influence tumor-immune system interactions.

To further characterize this behavior, we now establish the necessary conditions under which a Bogdanov-Takens bifurcation occurs in the system (1).

#### Theorem 2.4

The set

11 $$\begin{aligned} B = \{ (b,r,\alpha ,\gamma ,\beta ,\rho ,\delta ,\eta , \omega ,\lambda ) \mid BT = 0 \} \end{aligned}$$

contains the Bogdanov-Takens bifurcations of system (1).

#### Proof

We consider the system (1), its Jacobian matrix (4), and its characteristic polynomial (10). To obtain two zero roots in the characteristic polynomial (10), we must satisfy conditions $a_0 = a_1 = 0$. Under this assumption, the polynomial takes the form:

$$\begin{aligned} P(\psi ) = \psi ^2 (\psi - a_2), \end{aligned}$$

whose solutions consist of two zero eigenvalues and a single nonzero real eigenvalue, for $a_{2}\ne 0$.

To determine the Bogdanov-Takens (BT) bifurcations, we first express the coefficients of the characteristic polynomial, $a_0 = \det (J)$ and $a_1 = \text {tr}(J)$, solely in terms of the cancer cell density *C*, utilizing the expressions for *M* (5) and *T* (6). The BT points are defined by the simultaneous satisfaction of $a_0=0$ and $a_1=0$. Specifically, by finding the common roots between $a_0$ and $a_1$ with respect to *C*, we obtain a single constraint on the system parameters, $BT=0$ by the method from Kuzovatov and Kytmanov (2025). The full expression for the constraint $BT=0$ is given by the sum of equations (35) and (36). $\square$

The principal results of the bifurcation analysis for the complete model (1) are summarized in the bifurcation diagram shown in Figure 11.

## Simplified Model

The full model (1) describes the dynamics of cancer cells $(C)$, T cells $(T)$, and monocytes $(M)$, with monocytes mediating the immunosuppressive feedback exploited by tumor cells. To reduce model complexity and enable detailed bifurcation analysis, we apply a quasi-steady-state approximation (QSSA) to the monocyte population. Specifically, we assume that monocytes reach equilibrium on a faster timescale relative to the cancer and T cell populations, i.e., $\frac{dM}{dt} \approx 0$. Solving the algebraic equation

$$\begin{aligned} \rho - \eta M - \omega C M = 0, \end{aligned}$$

then

12 $$\begin{aligned} M = \frac{\rho }{\eta + \omega C}, \end{aligned}$$

and substituting into the T cell equation yields the reduced system (13), where the monocyte-mediated effect is incorporated through a nonlinear, saturating term in the growth of $T$.

This reduction is biologically meaningful under the assumption that monocyte dynamics are significantly faster due to high turnover or rapid regulation, consistent with immunological observations. Supporting this assumption, the literature indicates that circulating monocytes have a very short lifespan. In murine models, classical monocytes have a half-life of approximately 20–24 hours, whereas in humans, they can disappear from circulation within less than a day (Song et al. 2016; Patel et al. 2017; Teh et al. 2019). In contrast, T cells are markedly more long-lived. Naive T cells, for example, can persist for the entire lifetime of the host, with some studies suggesting a lifespan of up to 100 years (Nasi et al. 2006; Rangel Rivera et al. 2021). These temporal differences provide strong biological justification for applying a quasi-steady-state approximation to the monocyte population, as their dynamics occur on a significantly faster timescale than those of tumor or T cell populations. Nevertheless, it should be noted that this approximation inevitably neglects transient behaviors and feedback loops arising from the dynamic response of $M$, and may alter the position or stability of bifurcation structures, particularly near codimension-two points where all variables interact strongly. The quasi-steady-state relation (12) employed above corresponds to the fast equilibration of the monocyte compartment under the assumption that monocyte dynamics are much faster than those of $T$ and $C$. Under this approximation $M(t)$ is taken to track the instantaneous tumor load $C(t)$: for instance, when $C=0$ the fast equilibrium yields $M=\rho /\eta$, whereas an increase in $C$ reduces the fast equilibrium value via the factor $\eta +\omega C$. Thus the QSSA implies that $M$ responds essentially to the current value of $C$ and does not retain detailed memory of which we shall denote by ast tumour dynamics. If the maturation stage has a transition rate denoted by $\sigma$, the QSSA remains valid only when the maturation timescale is sufficiently short, i.e. $1/\sigma \ll 1/\delta$ and $1/\sigma \ll 1/r$. If these inequalities are not satisfied, algebraic elimination becomes inappropriate, leading to maturation-induced dynamical effects, which may include delay-induced oscillations or altered bifurcation loci.

Substituting the expression in (12) into the second equation of system (1), we obtain the following reduced model:

13 $$\begin{aligned} \begin{array}{lcl} \frac{d C}{d t} & =& r C \left( 1 - b C\right) \left( C-\gamma \right) - \alpha C T,\\ \frac{dT}{dt} & =& \lambda C + \dfrac{\beta \rho }{\eta + \omega C} T - \delta T.\\ \end{array} \end{aligned}$$

The reduced model can capture the essential characteristics of the interaction between cancer and the immune system and preserves the qualitative behavior of the bifurcation landscape, as described in Fenichel (1979), Jones (1995), and Kuehn (2015). This balance between manageability and biological fidelity justifies its use for analytical exploration.

This reduced system captures the essential nonlinear interactions between tumor cells and the immune response, while incorporating the immunomodulatory role of monocytes through the parameter-dependent term as:

$$\begin{aligned} \beta M(C) \;=\; \frac{\beta \,\rho }{\eta + \omega \,C}, \end{aligned}$$

that represents the capacity of monocytes to stimulate cytotoxic T lymphocytes (CTLs). This stimulatory potential decreases as the tumor burden $C$ increases—reflecting the suppressive effect $\omega \,C$ exerted by cancer cells, remains constrained by the basal monocyte turnover rate $\eta$, and is driven by the monocyte recruitment rate $\rho$ and the activation efficiency $\beta$. Consequently, the term $\beta M(C)$ succinctly captures how monocytes, whose functionality is modulated by the presence of tumor cells, in turn regulate T cell expansion within the tumor microenvironment.

The term $\beta M(C)$ not only quantifies the strength with which monocytes stimulate cytotoxic T lymphocytes (CTLs), but also directly explains how the tumor can evade the immune response, as $C$ increases, the denominator $\eta + \omega \,C$ grows so that

$$\begin{aligned} M(C) \;=\;\frac{\rho }{\eta + \omega \,C} \;\longrightarrow \;0 \quad \text {as }C\rightarrow \infty , \end{aligned}$$

leading to a collapse of $\beta M(C)$ and insufficient CTL activation, furthermore, any further reduction in $\beta$ (for instance, due to loss of $\beta _2$-microglobulin or blockade of MHCI cross-dressing) drives $\beta M(C)$ below the critical threshold, shifting the system into a tumor-escape regime. In sum, $\beta M(C)$ compactly captures how the tumor, by suppressing monocytes (via $\omega C$) and/or reducing $\beta$, disables CTL expansion and thus evades immune surveillance.

This reduced model (13) allows for the analysis of tumor-immune interactions under the assumption of a fast timescale for Monocyte dynamics, effectively decoupling them from the system.

Now if we solve the second equation in (13) for the T-Cells

14 $$\begin{aligned} T = \frac{\lambda (\eta + \omega C)C}{\delta (\eta + \omega C) - \beta \rho }. \end{aligned}$$

**Fig. 1:**
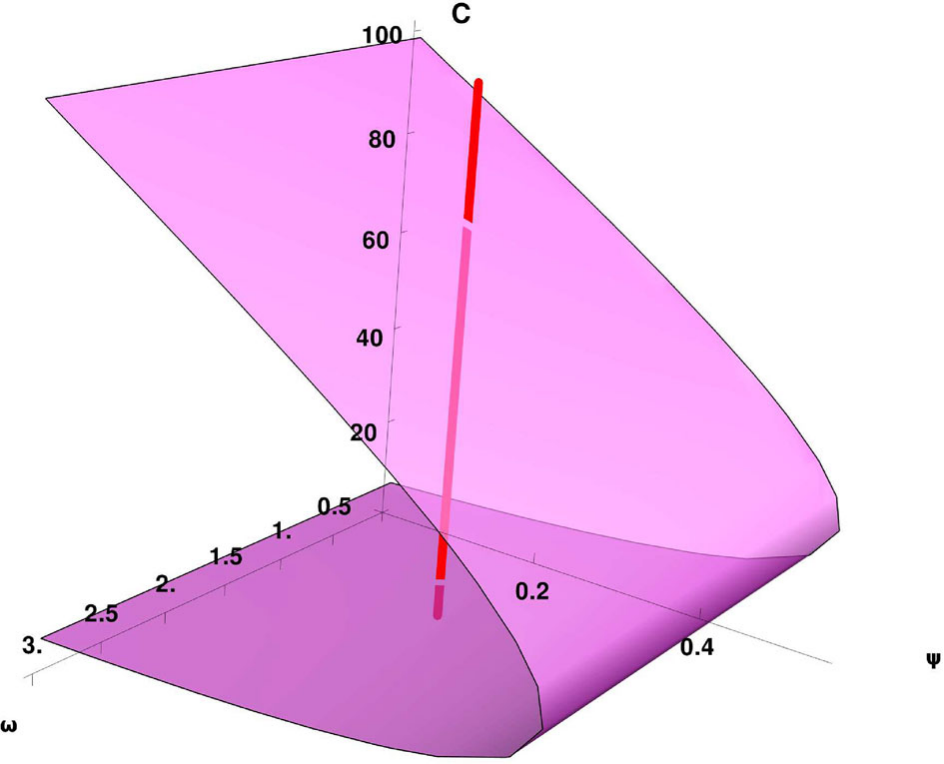
Critical surface in the $(\omega , \psi , C)$ space, where the tumor-immune dynamics reach equilibrium. The parameter $\omega$ reflects the inhibitory effect of tumor cells on monocytes, and $\psi$ captures the relative strength of the immune response. The red vertical line highlights two biologically relevant positive steady states. The parameters are as follows: $b=0.01$, $\gamma = 7.17$, $\delta = 1$, $\rho =0$, $\lambda =0.2$, $\eta =0$, and $\beta =0.015$

Substituting the equilibrium value of $T$ into the first equation of system (13), we obtain an expression for the critical points of the system as a function of the tumor cell population $C$ only. This leads to the following equation:

15 $$\begin{aligned} \psi = \frac{(1 - b C)(C - \gamma )\left( \delta (\omega C + \eta ) - \beta \rho \right) }{(\omega C + \eta )C}, \end{aligned}$$

where $\psi = \frac{\alpha \lambda }{r}$ is a positive, dimensionless parameter that combines key biological effects: $\lambda$ represents the antigenicity, i.e., the efficiency with which tumor cells stimulate the immune response; $\alpha$ denotes the rate at which tumor cells are eliminated upon interaction with T cells; and $r$ is the intrinsic proliferation rate of tumor cells. The vertical red line in Figure 1 represents a parameter slice in the $(\omega ,\psi )$–space rather than a locus connecting the boundary points (*C*, 0) and (0, *T*). The surface shown in the figure corresponds to the branch of positive, non-trivial equilibria expressed as a function of the tumor variable *C*. A saddle-node bifurcation occurs when the red vertical line becomes tangent to this surface, at which point two non-trivial steady states collide and disappear. Since $\psi$ is defined using the non-trivial stationary solution for *T* in equation (14), the pair (*C*, 0) cannot belong to this branch unless $C=0$. Moreover, expression (15) requires $C\ne 0$ for the definition of $\psi$.

The parameter $\psi$ can be interpreted as an *effective immune response index*: it quantifies the relative strength of the immune system in targeting and eliminating tumor cells compared to the tumor’s growth capacity. A higher value of $\psi$ suggests a more dominant immune response capable of controlling or eliminating the tumor, while a lower $\psi$ indicates a potentially weaker immune control, allowing the tumor to grow unchecked. Figure 1 illustrates the critical points of the system as a function of the tumor cell population $C$. The surface quantifies how the tumor-mediated suppression of monocytes, represented by $\omega$, and the effective immune response index, $\psi$, interact to determine system equilibria. The vertical red line marks the location of two positive critical points, indicating possible steady states of tumor presence under specific immune and suppressive conditions.

The origin $(C, T) = (0, 0)$ is always a critical point of the system, corresponding to the absence of both tumor and immune cell populations. Additional positive critical points $(C^*, T^*)$, with $C^* > 0$ and $T^* > 0$, may exist depending on the parameters. These points are determined by the solutions of the scalar function $\psi (C)$, which must admit real, positive roots for biologically meaningful equilibria to arise.

Depending on the values of the model parameters, the system may exhibit different dynamical regimes. A single positive equilibrium may arise when two critical points merge, typically through a saddle-node bifurcation, and it may correspond to a stable state representing either tumor elimination or coexistence. In other cases, the system may present two distinct positive equilibria, reflecting a bistable regime in which the long-term outcome—tumor persistence or eradication—depends sensitively on initial conditions and parameter configurations. In contrast, the absence of positive equilibria indicates that the immune response is unable to counteract tumor expansion, leading to uncontrolled tumor growth, commonly termed the escape phase. The following result is analogous to the Proposition 2.1.

### Proposition 3.1

(Stability of the trivial equilibrium) The origin $(C, T) = (0, 0)$ is always a critical point in the system. The stability of this point depends on the relation between the immune response parameter $\beta \rho / \eta$ and the decay rate $\delta$ of T-cells:

(i) If $\displaystyle \frac{\beta \rho }{\eta } < \delta$, the origin is a locally asymptotically stable node.
(ii) If $\displaystyle \frac{\beta \rho }{\eta } > \delta$, the origin is a saddle point and hence unstable.

### Proof

The Jacobian matrix evaluated at the origin $(C, T) = (0, 0)$ is:

$$\begin{aligned} J(0, 0) = \begin{pmatrix} -r \gamma & 0 \\ \lambda & -\delta + \dfrac{\beta \rho }{\eta } \end{pmatrix}. \end{aligned}$$

This matrix has two real eigenvalues:

$$\begin{aligned} \lambda _1 = -r \gamma < 0, \quad \lambda _2 = -\delta + \frac{\beta \rho }{\eta }. \end{aligned}$$

The sign of $\lambda _2$ determines the stability of the origin. If $\lambda _2 < 0$, both eigenvalues are negative and the equilibrium is stable. If $\lambda _2 > 0$, the equilibrium is a saddle point due to one positive eigenvalue. $\square$

### Proposition 3.2

Let $(C^*, T^*)$ be a positive equilibrium point of the reduced system (13), with $C^* > 0$ and $T^* > 0$. The local stability of this equilibrium is determined by the sign of the trace and determinant of the Jacobian matrix evaluated at $C^*$, given by:

16 $$\begin{aligned} \textrm{tr}(C^*)&= -r \left[ C^*(-2b\gamma + 3b C^* - 2) + \gamma \right] - \delta - \frac{\alpha \lambda C^*(\eta + C^* \omega )}{-\beta \rho + \delta \eta + \delta C^* \omega } + \frac{\beta \rho }{\eta + C^* \omega }, \end{aligned}$$

17Then, the critical point $(C^*, T^*)$ is:

- **Locally asymptotically stable** if $\textrm{tr}(C^*) < 0$ and $\textrm{det}(C^*) > 0$,
- **A saddle point (unstable)** if $\textrm{det}(C^*) < 0$,
- **Unstable node or focus** if $\textrm{tr}(C^*) > 0$ and $\textrm{det}(C^*) > 0$,
- **Non-hyperbolic** if $\textrm{det}(C^*) = 0$, in which case linear analysis is inconclusive.

### Proof

The local stability of a planar dynamical system is determined by the eigenvalues of the Jacobian matrix evaluated at the equilibrium point $(C^*, T^*)$ . The sign of the determinant and trace provide information about the nature of these eigenvalues: A negative determinant implies real eigenvalues of opposite signs, indicating a saddle point and thus instability. A positive determinant with negative trace implies that both eigenvalues have negative real parts, hence the equilibrium is locally asymptotically stable. A positive determinant with positive trace implies instability due to eigenvalues with positive real parts. A zero determinant indicates a non-hyperbolic equilibrium, and linear stability analysis is not sufficient.

Equations (16) and (17) are obtained by computing the Jacobian matrix of the reduced system and evaluating it at $(C^*, T^*)$, taking into account the expression of $T^*$ in terms of $C^*$. Thus, the result follows from standard planar dynamical systems theory. $\square$

The stability analysis of the positive equilibria $(C^*, T^*)$ reveals biologically meaningful insights into the dynamic interplay between tumor cells, the adaptive immune response, and the modulatory role of monocytes:

A *locally asymptotically stable* equilibrium corresponds to a controlled or dormant tumor state, where T cells maintain the cancer population at a manageable level. In this regime, the monocyte population—assumed to be in quasi-equilibrium—continuously shapes the immune response via cytokine signaling and antigen presentation. This scenario may reflect remission or clinical stability. A *saddle point* indicates a bistable regime in which the system may either eliminate the tumor or allow its progression, depending on initial conditions or external perturbations (e.g., immunotherapy). Monocytes, by influencing T cell activation through the parameter $\omega$, can tip the balance toward either tumor control or immune escape, suggesting their potential as therapeutic targets. An *unstable equilibrium* signifies that the tumor dominates the immune system, leading to unchecked cancer growth. In this phase, monocytes may be reprogrammed by the tumor microenvironment into tumor-associated macrophages (TAMs), which can suppress T cell activity and promote tumor progression and invasion, highlighting a critical oncological feedback mechanism. If the equilibrium is *non-hyperbolic*, the system may be near a bifurcation point, where small changes in parameters can lead to qualitative shifts in outcomes. The equilibrium structure not only reflects immune efficacy but also encapsulates the essential role of monocytes as intermediaries that can either enhance immune surveillance or facilitate immune evasion, making them key players in the tumor–immune landscape from both a biological and oncological perspective.

## Bifurcation Analysis in Reduced Model

To better understand the qualitative transitions in the tumor–immune dynamics, we explore the bifurcation structure of the reduced system (13). In particular, we study how variations in biologically relevant parameters affect the number and stability of equilibrium points.

### Equilibrium Bifurcations and Tumor Outcomes

As derived in Equations (7) (15), the condition

$$\begin{aligned} \psi = \frac{(1 - b C)(C - \gamma )(\delta (\eta + C\omega ) - \beta \rho )}{C(\eta + C\omega )}, \end{aligned}$$

characterizes the set of critical points as a function of the cancer cell population $C$, where $\psi = \alpha \lambda / r$ or $\Gamma = \alpha \lambda / r$ is a dimensionless bifurcation parameter incorporating immune efficiency and tumor growth dynamics. The number of real positive solutions to this equation determines the number of biologically meaningful equilibria. Depending on the shape of the right-hand side as a function of $C$, the system may exhibit:

- A unique positive equilibrium (monostability when two critical points collide and disapear by saddle-node bifurcation),
- Two positive equilibria (bistability), or
- No real or positive equilibrium.

These transitions correspond to *saddle-node bifurcations* in parameters when, two equilibria coalesce and vanish, changing the qualitative behavior of the system. These bifurcations can be visualized by plotting the function on the right-hand side of (15) and identifying values of $\psi$ where the number of positive intersections changes.

#### Proposition 4.1

(Saddle-node bifurcation)

The reduced system (13) exhibits a hypersurface of saddle-node bifurcations in the parameter space. This bifurcation set is characterized by the following condition:

18 $$\begin{aligned} {\mathcal {S}}_{r} = \left\{ (b, r, \alpha , \gamma , \beta , \rho , \delta , \eta , \omega , \lambda ) \big | \ SN_{r} = 0 \right\} , \end{aligned}$$

where $SN_r = 0$ defines the saddle-node bifurcation condition derived from the degeneracy of equilibria, and ${\mathcal {S}}_{r}$ represents the set of all parameter combinations for which two positive steady states coalesce into a non-hyperbolic equilibrium.

#### Proof

We analyze the algebraic condition that defines the cancer equilibrium $C^*$, given implicitly by equation (15), which is equivalent to

$$\begin{aligned} \alpha \lambda \, C(\eta + C \omega ) - r (1 - b C)(C - \gamma )\left[ \delta (\eta + C \omega ) - \beta \rho \right] = 0. \end{aligned}$$

By computing the discriminant of this expression with respect to the cancer population variable $C$, we obtain the hypersurface ${\mathcal {S}}_r$ in parameter space that characterizes the saddle-node bifurcation.^1^ The vanishing of the discriminant ensures the coalescence and annihilation of two equilibrium branches, which is the defining feature of a saddle-node bifurcation. $\square$

**Fig. 2:**
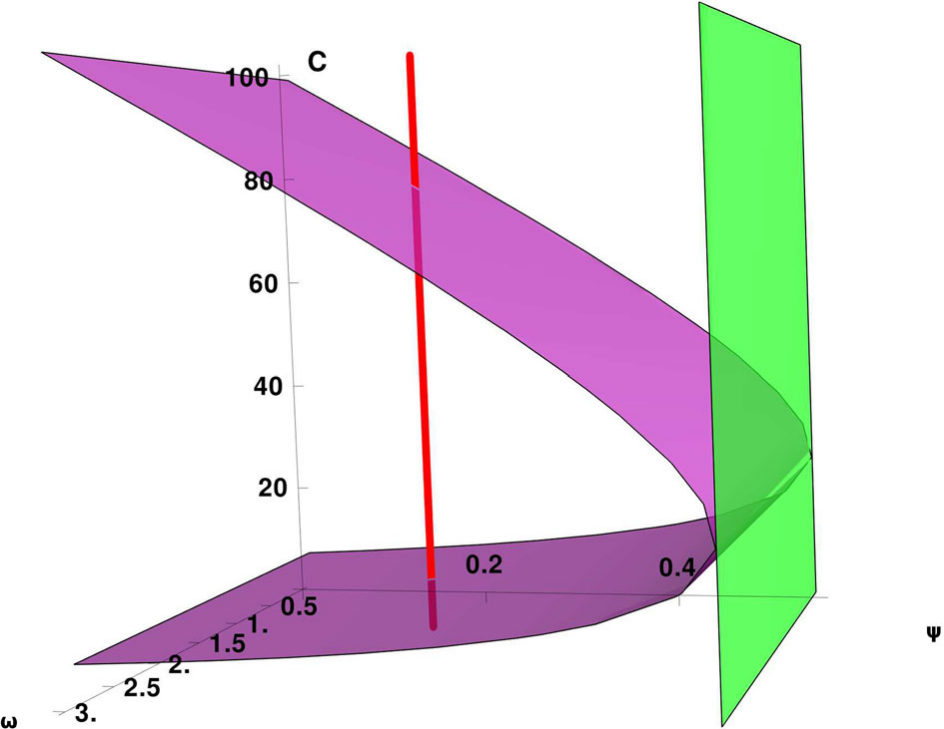
Bifurcation structure of the reduced system with respect to the effective immune activation parameter $\psi$. The purple surface represents critical points of the system, while the green surface corresponds to the saddle-node bifurcation hypersurface ${\mathcal {S}}_r$. Vertical red lines indicate transitions in the number of equilibria. Intersections of the surfaces mark parameter regimes with multiple steady states, revealing the presence of bistability. The parameters are as follows: $b=0.01$, $\gamma = 7.17$, $\delta = 1$, $\rho =0$, $\lambda =0.2$, $\eta =0$, and $\beta =0.015$

Figure 2 provides a visual representation of the saddle-node bifurcation set $S_{r}$ described in Proposition 4.1. The green surface corresponds to the parameter combinations for which the reduced system undergoes a saddle-node bifurcation, where two equilibria coalesce into a non-hyperbolic point. The purple surface shows the loci of critical points of the system, and their intersection with the green surface highlights the bifurcation points. Vertical red lines trace the variation of equilibria as a function of the effective immune activation parameter $\psi$, indicating regions with one or multiple steady states. This geometric visualization supports the analytical condition (18) and illustrates how changes in immune activation can induce bistability in the system.

Having characterized the saddle-node bifurcation set $S_{r}$, which identifies parameter combinations where equilibria merge and vanish, we now turn our attention to another fundamental codimension-one bifurcation: the Hopf bifurcation. Unlike saddle-node bifurcations, which are associated with changes in the number of steady states, Hopf bifurcations signal the onset of oscillatory dynamics through the emergence of a limit cycle. In the context of our reduced system (13), this occurs when a pair of complex conjugate eigenvalues of the Jacobian matrix cross the imaginary axis. The following proposition formalizes the algebraic condition defining the hypersurface in parameter space where such a transition occurs.

#### Proposition 4.2

(Hopf bifurcation)

The reduced system (13) exhibits a hypersurface of Hopf bifurcations in the parameter space. This bifurcation set is characterized by the following condition:

19 $$\begin{aligned} {\mathcal {H}}_{r} = \left\{ (b, r, \alpha , \gamma , \beta , \rho , \delta , \eta , \omega , \lambda ) \big | \ Hopf_{r} = 0 \right\} , \end{aligned}$$

where $\textrm{Hopf}_r = 0$ defines the hypersurface in parameter space associated with Hopf and symmetric-saddle bifurcations.

#### Proof

To calculate the hypersurface in parameters, we get the reduced model (13) and we calculate the Jacobian matrix *A*. Then we have the equations of trace (16), and we take the numerator as follows.

$$\begin{aligned} trA^{N}=\beta \rho - (C\omega + \eta ) (r (C (-2 b\gamma + 3 b C - 2) + \gamma ) + \delta + \alpha T). \end{aligned}$$

On the other hand, we get the first and second equation from the system (13) as

$$\begin{aligned} f_{1}(C,T)= &  r C \left( 1 - b C\right) \left( C-\gamma \right) - \alpha C T,\\ f_{2}(C,T)= &  \lambda C + \dfrac{\beta \rho }{\eta + \omega C} T - \delta T\\= &  \frac{C\lambda (C\omega + \eta ) - \delta T (C\omega + \eta ) + \beta \rho T}{\eta + \omega C}\\ \end{aligned}$$

also in terms of parameters. In order to calculate the Sylvester determinant matrix (Parker 1935; Woody 2016; Kuzovatov and Kytmanov 2025) to find roots in common between two polynomials, we only take the numerator of $f_{2}(C,T)$ as $f_{2}^{N}(C,T)$. Then we perform the Sylvester determinant matrix between $f_{1}(C,T)$ and $trA^{N}$, with respect to *T*, and we get

$$\begin{aligned} SD(C)_{1}=(C\omega + \eta ) (C r (-b\gamma + 2 b C - 1) + \delta ) - \beta \rho . \end{aligned}$$

On the other hand, we calculate the Sylvester determinant matrix between $f_{2}(C,T)^{N}$ and $trA^{N}$, with respect to *T* too, and get

$$\begin{aligned} SD(C)_{2}= &  \beta ^2 \rho ^2 + r (C (-2 b\gamma + 3 b C - 2) + \gamma ) (C\omega + \eta ) (\delta (C\omega + \eta ) - \beta \ \rho )\\ &  + \alpha C\lambda (C\omega + \eta )^2 - 2\beta \delta \rho (C\omega + \eta ) + \delta ^2 (C\omega + \eta )^2. \end{aligned}$$

Lastly we perform the Sylvester determinant matrix between $SD(C)_{1}$ and $SD(C)_{2}$ respect to *C*, and we obtain the hypersurface ${Hopf}_r$^2^ in parameters. $\square$

Following the characterization of the Hopf bifurcation set, we now turn our attention to a more intricate bifurcation phenomenon that encapsulates the interaction between both saddle-node and Hopf mechanisms—namely, the Bogdanov–Takens (BT) bifurcation. This codimension-two bifurcation arises when an equilibrium point simultaneously satisfies the conditions of a zero eigenvalue (associated with a saddle-node) and a pair of purely imaginary eigenvalues (associated with a Hopf bifurcation). The BT bifurcation serves as an organizing center in the dynamics of nonlinear systems, leading to the emergence of rich local behaviors. In the context of our reduced model, we analytically derive the hypersurface in parameter space corresponding to the BT bifurcation condition.

#### Theorem 4.3

(Bogdanov–Takens bifurcation)

The reduced system (13) exhibits a hypersurface of Bogdanov–Takens (BT) bifurcations in the parameter space.

**(i)**: The set of parameter combinations at which the system undergoes a Bogdanov–Takens bifurcation is given by:
  20 $$\begin{aligned} {\mathcal {B}}_{r} = \left\{ (b, r, \alpha , \gamma , \beta , \rho , \delta , \eta , \omega , \lambda ) \big | \ BT_{r} = 0 \right\} , \end{aligned}$$
  where $BT_r = 0$ denotes the defining condition of the codimension two of Bogdanov–Takens bifurcation.
**(ii)**: A degenerate Bogdanov–Takens bifurcation occurs in the point
  21 $$\begin{aligned} \left( T^*,C^{*}\right)= &  \sqrt{\frac{\gamma }{b}}\left( \frac{r \left( \sqrt{b \gamma } - 1 \right) ^2}{\alpha } , 1 \right) \nonumber \\ Deg= &  \left\{ \beta \rho = \delta \eta , \eta = 0, \alpha \lambda = \delta r \left( \sqrt{b \gamma } - 1 \right) ^2, \right. \nonumber \\ &  \left. \delta \sqrt{b} = b \gamma ^{3/2} r - 2 \sqrt{b} \gamma r + r \sqrt{\gamma } \right\} . \end{aligned}$$

#### Proof

(*i*) To obtain the Bogdanov–Takens bifurcation, we consider the reduced model (13) and compute its Jacobian matrix *A*. By substituting the expression for

$$\begin{aligned} T=\frac{\lambda (C\omega + \eta )C}{\delta (C\omega + \eta ) - \beta \rho } \end{aligned}$$

into the trace and determinant of *A*, we obtain analytical expressions in terms of the model parameters and the variable *C*, and only take the numerator in order to calculate the Sylvester determinant matrix, as follows.

$$\begin{aligned} trA(C)^{N}= &  -\beta ^2 \rho ^2 - r (C (-2 b\gamma + 3 b C - 2) + \gamma ) (C\omega + \eta ) (\delta (C\omega + \eta ) - \beta \ \rho )\\ &  - \alpha C\lambda (C\omega + \eta )^2 + 2\beta \delta \rho (C\omega + \eta ) - \delta ^2 (C\omega + \eta )^2,\\ detA(C)^{N}= &  r (T (-2 b\gamma + 3 b T - 2) + \gamma ) (\beta \rho - \delta (\eta + T\omega ))^2 + 2 \alpha \lambda T\delta (\eta + T\omega )^2\\ &  - \alpha \lambda \beta \rho T (2\eta + 3 T\omega ). \end{aligned}$$

The common roots of these expressions are found by computing the determinant of the Sylvester matrix in terms of *C*. This procedure yields the parameter condition $BT_r$^3^ that characterizes the bifurcation.

(*ii*) To prove the non-degeneracy, we use the projection method as described in Kuznetsov (2005) to compute the coefficients $a_{2}$ and $b_{2}$ of following normal form,

22 $$\begin{aligned} \begin{array}{lcll} \dot{\xi _{0}}& =& \xi _{1},\\ \dot{\xi _{1}}& =& \beta _{1}+\beta _{2}\xi _{0} + a_{2} \xi _{0}^2 + b_{2} \xi _{0}\xi _{1}, \end{array} \end{aligned}$$

and we will show that the condition $a_{2} \cdot b_{2} \ne 0$ is satisfied. The critical point

$$\begin{aligned} (C^{*},T^{*})=\sqrt{\frac{\gamma }{b}}\left( \frac{r\left( \sqrt{b\gamma } - 1 \right) ^2}{\alpha },1\right) \end{aligned}$$

satisfies the saddle-node and Hopf condition under the following parameter combination

$$\begin{aligned} \left\{ \beta \rho = \delta \eta , \eta = 0, \alpha \lambda = \delta r \left( \sqrt{b \gamma } - 1 \right) ^2, \delta \sqrt{b} = b \gamma ^{3/2} r - 2 \sqrt{b} \gamma r + r \sqrt{\gamma } \right\} . \end{aligned}$$

Then, the Jacobian matrix evaluated at that point $(C^{*},T^{*})$ has the following form.

$$\begin{aligned} A=\kappa \left( \begin{array}{cc} 1 & - \frac{\alpha }{\kappa }\sqrt{\frac{\gamma }{b}} \\ \frac{ r\left( \sqrt{b\gamma } - 1 \right) ^2}{\alpha } & 1 \\ \end{array} \right) , \end{aligned}$$

where $\kappa =r\left( \sqrt{b\gamma } - 1 \right) ^2\sqrt{\frac{\gamma }{b}}$, that is similar to the Jordan block form

$$\begin{aligned} \left( \begin{array}{cc} 0 & 1 \\ 0 & 0 \\ \end{array} \right) . \end{aligned}$$

In this case the generalized eigenvectors are

$$\begin{aligned} \left\{ q_{0},q_{1}\right\} =\left\{ \left( \begin{array}{c} \frac{\alpha \sqrt{\frac{b}{\gamma }}}{\kappa }\\ 1 \\ \end{array} \right) ,\left( \begin{array}{c} \frac{\alpha \sqrt{\frac{b}{\gamma }}}{\kappa ^2} \\ 0\\ \end{array} \right) \right\} , \hspace{0.1cm}\left\{ p_{0},p_{1}\right\} =\left\{ \left( \begin{array}{c} -\frac{\sqrt{\frac{b}{\gamma }}}{\alpha } \\ 0 \\ \end{array} \right) ,\left( \begin{array}{c} -\frac{\kappa \sqrt{\frac{\gamma }{b}}}{\alpha } \\ 1 \\ \end{array} \right) \right\} . \end{aligned}$$

For the second order terms of the system (13), we obtain the bi-linear form

23 $$\begin{aligned} &  B({\hat{u}},{\hat{v}})\nonumber \\ &  \quad = \left( \begin{matrix} 2 r u_{1} v_{1}\left( b\gamma - 3\sqrt{b\gamma } + 1 \right) - \alpha (u_{1}v_{2} + u_{2}v_{1})\\ 0 \end{matrix}\right) . \end{aligned}$$

We get the following coefficients for $a_{2}=\frac{1}{2}\langle p_{1}, B(q_{0},q_{0})\rangle$ and $b_{2}=\langle p_{0}, B(q_{0},q_{0})\rangle +\langle p_{1}, B(q_{0},q_{1})\rangle$, these coefficients are expressed in terms of the parameters system. We derive the expressions for $a_{2}$ and $b_{2}$ as follows:

$$\begin{aligned} a_{2}= &  -\frac{\alpha \gamma r^2\left( \sqrt{b\gamma } - 1 \right) ^2}{\sqrt{\alpha ^2 + r^2\left( \sqrt{b\gamma } - 1 \right) ^4}}\\ b_{2}= &  \frac{\alpha r\left( b\gamma - 4\sqrt{b\gamma } + 1 \right) }{\sqrt{\alpha ^2 + r^2\left( \sqrt{b\gamma } - 1 \right) ^4}}, \end{aligned}$$

For

$$\begin{aligned} \gamma =\frac{7 - 3\sqrt{5}}{2 b}, \end{aligned}$$

we have $a_{2}=-\frac{2\left( 4\sqrt{5} - 9 \right) \alpha r^2}{b\sqrt{4\alpha ^2 + \left( 14 - 6\sqrt{5} \right) r^2}}>0$, and $b_{4}=4 \alpha b r$, so $a_{2} b_{4} >0$. According to Bazykin et al. (1985); Berezovskaya and Khibnik (1985); Dumortier et al. (1987), a generic three-parameter unfolding of (13) is locally topologically equivalent to the canonical family:

24 $$\begin{aligned} \begin{array}{lcll} \dot{\xi _{0}}& =& \xi _{1},\\ \dot{\xi _{1}}& =& \beta _{1} +\beta _{2} \xi _{1}+\beta _{3} \xi _{0} \xi _{1} + a_{2} \xi _{0}^2 + b_{4} \xi _{0}^{3}\xi _{1}. \end{array} \end{aligned}$$

On the other hand, if we have $\gamma =1/b$, then $b_{2}=-2 r$ but $a_{2}=a_{3}=0$, so, the Bogdanov-Takens scenario is considered to be degenerate too. $\square$

From (13), the tumor growth function can be written as:

$$\begin{aligned} f(C) = r(1 - bC)(C - \gamma ), \end{aligned}$$

with roots at $C = \gamma$ (Allee threshold) and $C = \frac{1}{b}$ (saturation limit).

In the particular case when $\gamma = \frac{1}{b}$, the two roots coincide, and the expression reduces to:

25 $$\begin{aligned} f(C) = -\frac{r}{b}(1 - bC)^2, \end{aligned}$$

so the full dynamical equation becomes:

26 $$\begin{aligned} \frac{dC}{dt} = -\frac{r}{b} C(1 - bC)^2 - \alpha C T. \end{aligned}$$

This formulation reveals that the net growth rate is strictly negative for all $C > 0$, except at $C = \frac{1}{b}$, where it vanishes but also has zero derivative. Thus, under this extreme condition, the system prohibits tumor establishment: any initial presence of cancer cells inevitably leads to extinction, even in the absence of an immune response ($T = 0$).

This scenario represents a structural degeneration in which the minimal threshold for tumor survival ($\gamma$) coincides with the maximum population supported by the environment ($1/b$). Biologically, it corresponds to a microenvironment so hostile that tumor growth is fundamentally unsustainable.

**Fig. 3:**
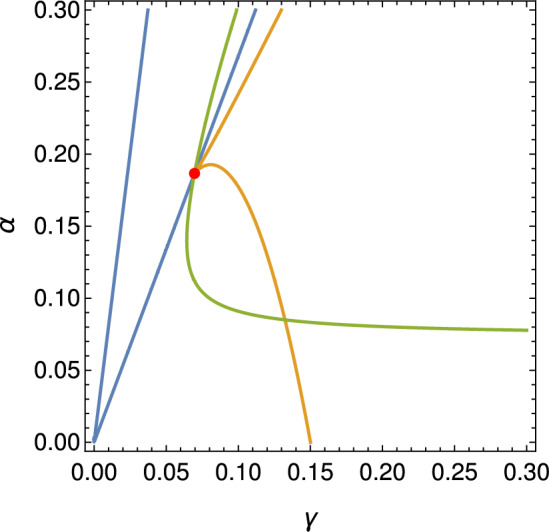
The red point marks a cusp-type Bogdanov–Takens bifurcation. The blue curves represent saddle-node bifurcations, the yellow curves correspond to Hopf-Symmetric-Saddles bifurcations, and the green curves indicate the Bogdanov–Takens bifurcation set, as detailed in Appendix A. The parameters are as follows : $$b = 0.01 $$, $$\gamma = 7 $$, $$\delta = 1 $$, $$ \rho = 3 $$, $$\lambda = 0.2 $$, $$\omega = 1 $$, $$\eta = 0$$, $$\beta = 0.015900262162975$$, $$r = 0.06975513690351436$$, and $$\alpha =0.18829545657252558$$

Mathematically, the case $\gamma = \frac{1}{b}$ corresponds to the collapse of multiple roots along with the vanishing of successive derivatives, giving rise to a highly degenerate and structurally unstable singularity within the unfolding of bifurcations. When $\gamma = \frac{7 - 3 \sqrt{5}}{2 b}$ we get a BT-degenerate of co-dimension three type *cusp*, and the associated geometry and dynamics of this case are illustrated in Figure 3.

Having characterized the codimension-two Bogdanov–Takens bifurcation, we now turn our attention to the Bautin bifurcation. This bifurcation arises when a Hopf bifurcation undergoes a qualitative change in its criticality, transitioning from supercritical to subcritical (or vice versa). Such a scenario occurs when the first Lyapunov coefficient vanishes, necessitating the computation of higher-order terms to determine the nature of the bifurcation. The Bautin bifurcation is thus a codimension-two bifurcation that refines the local dynamics near a Hopf point and serves as an organizing center for complex behaviors, including the coexistence of limit cycles. In the following theorem, we characterize the set of parameters where this degenerate Hopf (Bautin) bifurcation occurs.

#### Theorem 4.4

The following set

27 $$\begin{aligned} \text {Bautin}=\left\{ (b, r, \alpha , \gamma , \beta , \rho , \delta , \eta , \omega , \lambda ) \big | \sigma _{Bau}=0 \right\} \end{aligned}$$

contains the Bautin bifurcation in model (13).

#### Proof

Consider the model (13) and we get the first and second ordinary differential equations as *f*(*C*, *T*) and *g*(*C*, *T*), and the Jacobian matrix as *A*. By Chow et al. (1994), we find the first Lyapunov coefficient, and then we have

$$\begin{aligned} l_{1}= &  \frac{f_{T}}{(16 \omega _2^2)} (\omega _2 ( f_{T} (f_{CCC} + g_{CCT}) + 2 g_{T} (f_{CCT} + g_{CTT}) - g_{C} (f_{CTT} + g_{TTT}))\\ &  - f_{T} g_{T} (f_{CC}^2 - f_{CC} g_{CT} - f_{CT} g_{CC} - g_{CC} g_{TT} - 2 g_{CT}^2)\\ &  - g_{C} g_{T} (g_{TT}^2 - g_{TT} f_{CT} - g_{CT} f_{TT} - f_{CC} f_{TT} - 2 f_{CT}^2)\\ &  + f_{T}^2 (f_{CC} g_{CC} + g_{CC} g_{CT}) - g_{C}^2 (f_{TT} g_{TT} + f_{CT} f_{TT}) \\ &  - (\omega _2 + 3 g_{T}^2) (f_{CC} f_{CT} - g_{CT} g_{TT}) ), \end{aligned}$$

where $\omega _{2}=\det (A)$ and $f_{CT}=\frac{\partial ^{2} f}{\partial C \partial T}\mid _{T=T^{*}(C)}$,...etc., and $T^{*}$ as in (6). Under this expression, we have the following condition in terms of variable *C* and parameters.

$$\begin{aligned} L_{1}= &  -\beta ^4 \rho ^4 + \beta C^3 \rho r \omega ^3 \Bigg ( \delta \Big ( 2 C r \Big ( -18 b^2 C^3 + b \gamma ^2 + 15 b C^2 (b \gamma + 1)\\ &  - 4 C (b \gamma + 1)^2 + \gamma \Big ) \\ &  + \delta \Big ( C (8 b \gamma - 27 b C + 8) + \gamma \Big ) + \alpha C^2 \lambda \big ( b (\gamma + 6 C) + 1 \big ) \Big ) \Bigg ) \\ &  + C^5 \delta \omega ^4 \Bigg ( \delta r \Big ( 18 b^2 C^3 r - 15 b C^2 r (b \gamma + 1) + 9 b C \delta + 4 C r (b \gamma + 1)^2 \\ &  - (b \gamma + 1)(3 \delta + \gamma r) \Big ) \\ &  + \alpha \lambda (\delta - 2 C (b \gamma r + r)) \Bigg ) \\ &  + \beta ^2 C^2 \rho ^2 \omega ^2 \Bigg ( 18 b^2 C^4 r^2 - 15 b C^3 r^2 (b \gamma + 1) + C^2 r \Big ( 27 b \delta + 4 r (b \gamma + 1)^2 \Big ) \\ &  - C r (b \gamma + 1)(7 \delta + \gamma r) - \alpha C \lambda - \delta (\delta + 2 \gamma r) \Bigg ) \\ &  + \beta ^3 C \rho ^3 \omega \Big ( r \big ( C (2 b \gamma - 9 b C + 2) + \gamma \big ) + 2 \delta \Big ). \end{aligned}$$

Lastly, if we calculate the common root between *L*1 and *Tr*(*A*) with respect to variable *C*, we get the whole expression $\sigma _{Bau}$ that describes the Bautin bifurcation only in parameters. $\square$

A projection of $\sigma _{Bau}$ is found in (32) Appendix A.

#### Corollary 4.5

The following set

28 $$ \begin{aligned} Bau_{0}=\left\{ (b, r, \alpha , \gamma , \beta , \rho , \delta , \eta , \omega , \lambda ) \bigg | \ \omega =\frac{\Gamma _{2}+\Gamma _{3}}{\Gamma _{1}}, \beta \rho = \delta \eta , \, \& \, \eta =0 \right\} , \end{aligned}$$

where

$$\begin{aligned} \Gamma _{1}= &  16\sqrt{2} b^2 (b\gamma + 1) (b\gamma (5 b\gamma - 6) + 5),\\ \Gamma _{2}= &  -b\Lambda r (b\gamma (b\gamma - 14) + 1) (b\gamma (b\gamma - 6) + 1),\\ \Gamma _{3}^{2}= &  \left( 4 (b\gamma - 3) (3 b\gamma - 1) (b\gamma (5 b\gamma - 6) + 5)\left( b^2\gamma ^2 - 1 \right) ^2 \right. \\ &  \left. + (b\gamma (b\gamma - 14) + 1) (b\gamma \Lambda (b\gamma - 6) + \Lambda )^2 \right) \\ &  (b^2 r^2 (b\gamma (b\gamma - 14) + 1)),\\ \Lambda ^{2}= &  b\gamma (b\gamma - 14) + 1, \end{aligned}$$

characterizes the Bautin points of codimension 2 in system (13) at the critical point

$$\begin{aligned} \left( C^{**},T^{**}\right) =\frac{b\gamma + 1}{4 b}\left( 1,\frac{\lambda }{\delta }\right) . \end{aligned}$$

#### Proof

Consider the system (13) and the critical point $$\left( C^{**},T^{**}\right) $$. Computing the Jacobian matrix of the system (13) at $$ \left( C^{**},T^{**}\right) $$ yields:

$$\begin{aligned} A = \left( \begin{array}{cc}\frac{r (b\gamma + 1)^2}{8 b} & - \frac{\alpha + \alpha b\gamma }{4 b} \ \\ \frac{r^2 (b\gamma - 3) (b\gamma + 1) (3 b\gamma - 1)}{32\alpha b} & - \frac{r (b\gamma + 1)^2}{8 b} \\ \end{array} \right) . \end{aligned}$$

The eigenvalues of $A$ form a pair of complex conjugates, $\pm i \hat{\omega }$,

$$\begin{aligned} \hat{\omega }^{2} = \frac{r^2 (b\gamma + 1)^2 (b\gamma (b\gamma - 14) + 1)}{128 b}, \end{aligned}$$

with $(b\gamma (b\gamma - 14) + 1)>0$. The corresponding eigenvectors $p$ and $q$ satisfy:

$$\begin{aligned} Aq = i\omega q, \quad A^{T}p = -i \omega p. \end{aligned}$$

Then, the normalization condition $\langle p, q \rangle = 1$ holds if

$$\begin{aligned} p=\left( -\frac{r\left( 2 (b\gamma + 1) - i\sqrt{2 (b\gamma (b\gamma - 14) + 1)} \right) }{4\alpha },1\right) ^{T} \end{aligned}$$

and

$$\begin{aligned}q=\left( \frac{2\alpha \left( 2 (b\gamma + 1) + i\sqrt{2 (b\gamma (b\gamma - 14) + 1)} \right) }{r (b\gamma - 3) (3 b\gamma - 1)},1\right) ^{T}, \end{aligned}$$

with $\gamma \ne 3/b$ and $\gamma \ne 1/3b$. The multilinear functions $B(u,v)$ and $C(u,v,w)$ are given by:

$$\begin{aligned} B(u,v) = \left( \frac{1}{2} r u_1v_1 (b\gamma + 1) - \alpha (u_1v_2 + u_2v_1), \; 0 \right) , \end{aligned}$$

and

$$\begin{aligned} C(u,v,w) = \left( -6 b r u_1 v_1 w_1 , \; 0 \right) . \end{aligned}$$

The first Lyapunov coefficient $L_{1}(0)$ is computed as:

$$\begin{aligned} L_{1}(0)&= \frac{1}{2 \omega ^{2}} \, \textrm{Re} \left[ \left\langle p, C(q,q,{\bar{q}}) - 2 B\left( q, A^{-1} B(q,{\bar{q}})\right) \right. \right. \\&\quad \left. \left. + B\left( {\bar{q}}, (2 i \omega I_{2} - A)^{-1} B(q,q)\right) \right\rangle \right] \\&=-128 b^2\omega ^2 (b\gamma + 1) (-128 b^2\omega ^2 (b\gamma + 1) (b\gamma (5 b\gamma - 6) + 5))\\&\quad -128 b^2\omega ^2 (b\gamma + 1)( r^2 (b\gamma - 3) (b\gamma + 1)\\ &\qquad (3 b\gamma - 1) (b\gamma (b\gamma - 14) + 1) (b\gamma - 1)^2)\\&\quad +128 b^2\omega ^2 (b\gamma + 1) (8\sqrt{2} b\Lambda r\omega (b\gamma (b\gamma - 14) + 1) (b\gamma (b\gamma - 6) + 1)). \end{aligned}$$

If $\omega = (\Gamma _{2}+\Gamma _{3})/\Gamma _{1}$, then $L_{1}(0)=0$, with

$$\begin{aligned} \Gamma _{1}= &  16\sqrt{2} b^2 (b\gamma + 1) (b\gamma (5 b\gamma - 6) + 5),\\ \Gamma _{2}= &  -b\Lambda r (b\gamma (b\gamma - 14) + 1) (b\gamma (b\gamma - 6) + 1),\\ \Gamma _{3}^{2}= &  \left( 4 (b\gamma - 3) (3 b\gamma - 1) (b\gamma (5 b\gamma - 6) + 5)\left( b^2\gamma ^2 - 1 \right) ^2 \right. \\ &  \left. + (b\gamma (b\gamma - 14) + 1) (b\gamma \Lambda (b\gamma - 6) + \Lambda )^2 \right) \\ &  (b^2 r^2 (b\gamma (b\gamma - 14) + 1)),\\ \Lambda ^{2}= &  b\gamma (b\gamma - 14) + 1, \end{aligned}$$

To compute the second Lyapunov coefficient $L_2(0)$, we introduce the complex transformation $Y = z q + {\bar{z}} {\bar{q}}$ to derive the normal form in complex space:

$$\begin{aligned} z' = \lambda z + \sum _{l=2}^{4} G_l(z, {\bar{z}}) + O(\Vert z\Vert ^5). \end{aligned}$$

From this, we obtain the coefficients of $G_2(z, {\bar{z}})$ as $g_{02}, g_{11}, g_{20}$, and from $G_3(z, {\bar{z}})$, we obtain $g_{21}, g_{30}, g_{03}$. Similarly, from $G_4(z, {\bar{z}})$, we extract $g_{12}, g_{22}, g_{31}$. These coefficients are computed as

$$\begin{aligned} g_{ij} = \frac{1}{i! j!} \left( \frac{\partial ^{i+j} G_l}{\partial z^i \partial {\bar{z}}^j} \right) . \end{aligned}$$

The second Lyapunov coefficient $L_2(0)$ is given by

$$\begin{aligned} L_{2}(0)= &  \text {Re}\left[ g_{20}\right] + \frac{1}{\omega }\left( \text {Im}\left[ g_{20} \bar{g_{31}}-g_{11}\left( 4 g_{31}+ 3 \bar{g_{22}}\right) \right. \right. \\ &  \left. \left. -\frac{1}{2}g_{02}\left( g_{40}+\bar{g_{13}}\right) -g_{30}g_{12} \right] \right) \\= &  \frac{1}{\omega ^{2}} \left( \text {Re}\left[ g_{20}\left( \bar{g_{11}}\left( 3 g_{12}-\bar{g_{30}}\right) +g_{02} \left( \bar{g_{12}}-\frac{1}{3}g_{30}\right) +\frac{1}{3} \bar{g_{02}g_{03}}\right) \right] \right) \\ &  + \frac{1}{\omega ^{2}} \left( \text {Re}\left[ g_{11}\left( \bar{g_{02}}\left( \frac{5}{3}\bar{g_{30}}+ 3 g_{12}\right) +\frac{1}{3} g_{02} \bar{g_{03}}-4 g_{11}g_{30}\right) \right] \right. \\ &  \left. +\text {Im}\left[ g_{20} g_{11}\right] \text {Im}\left[ g_{21}\right] \right) \\= &  \frac{1}{\omega ^{3}} \left( \text {Im}\left[ g_{11} \bar{g_{02}}\left( \bar{g_{20}^{2}}-3 \bar{g_{20}}g_{11}- 4 g_{11}^{2}\right) \right] \right. \\ &  \left. + \text {Im}\left[ g_{20}g_{11}\right] \left( 3 \text {Re}\left[ g_{20} g_{11}\right] -2 g_{02} \bar{g_{02}}\right) \right) . \end{aligned}$$

The second Lyapunov coefficient $L_2$, associated with condition (28), can be formally expressed as a function of the system parameters. However, the resulting expression is a high-degree polynomial involving numerous nonlinear interactions, rendering a direct analytical interpretation intractable. To gain insight into the sign of $L_2$, numerical evaluation under fixed parameter values for $r$ and $\alpha$ indicates that $L_2 > 0$. Despite this, the structural complexity of the expression necessitates a more rigorous investigation to confirm the non-vanishing of $L_2$ in a neighborhood of the bifurcation point. $\square$

**Fig. 4:**
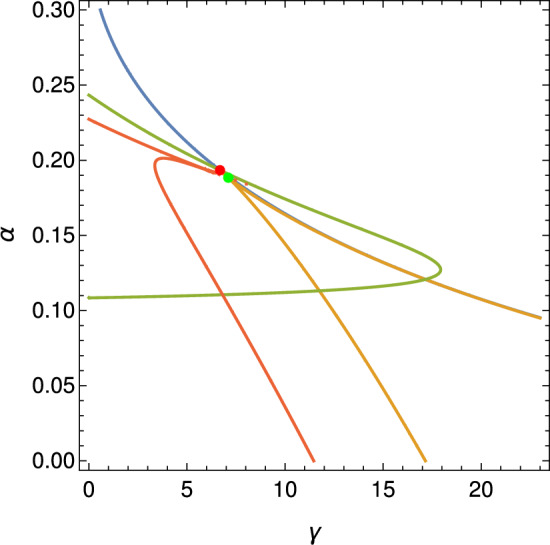
Bifurcation diagram corresponding to the analytical expressions presented in Appendix A. The color-coded curves represent distinct bifurcation sets: blue for saddle-node bifurcations, yellow for Hopf bifurcations, green for Bogdanov–Takens bifurcations, and red for the vanishing of the first Lyapunov coefficient. The red dot denotes the degenerate Bogdanov–Takens point $$\gamma =6.6842662$$ and $$\alpha =0.1933940$$, while the green dot indicates the Bautin bifurcation point of codimension two; $$\gamma =7.1067808$$ and $$\alpha =0.1883883$$. The parameters are as follows : $$b = 0.01 $$, $$\delta = 1 $$, $$ \rho = 0 $$, $$\lambda = 0.2 $$, $$\omega = 1 $$, $$\eta = 0$$, $$\beta = 0.015900262162975$$, and $$r = 0.0703553399686769$$

As illustrated in Figure 4, a numerical bifurcation diagram reveals the presence of a Bautin bifurcation, in agreement with the analytical condition established in Theorem 4.5. This diagram highlights the transition from supercritical to subcritical Hopf bifurcations and the emergence of a fold of limit cycles. In the subsequent section, we perform a numerical exploration to further validate and illustrate the analytical bifurcation results derived for the reduced system.

## Numerical Continuation

Numerical continuation methods complement the local analytical bifurcation analysis by enabling systematic computational tracing of solution branches as model parameters vary. In particular, continuation provides a way to verify and extend the existence of the bifurcations (saddle-node, Hopf, etc.) predicted by theory. In this way, numerical continuation validates the earlier bifurcation calculations in the mathematical oncology model and explores their implications over a broader range of parameters. In practice, we used continuation software MATCONT (Dhooge et al. 2008) to trace bifurcation curves in the parameter space and to monitor the stability of equilibrium branches. Starting from an equilibrium point, the algorithm follows the curve of such points as a second parameter is varied. Along these curves, changes in stability are detected automatically. The outcome is a detailed bifurcation diagram: saddle-node curves mark where equilibria appear or disappear, and Hopf curves indicate where oscillatory (limit-cycle) dynamics are born. These global bifurcation diagrams are essential for understanding the qualitative regimes of the tumor–immune model (13). By mapping out regions in parameter space corresponding to different dynamic behaviors (such as tumor clearance, tumor–immune coexistence, or immune escape), continuation analysis reveals how gradual changes in parameters can trigger abrupt regime shifts. For example, one may identify critical threshold curves of immune response strength or tumor growth rate that separate a stable tumor-free state from a persistent tumor state. Such knowledge helps predict the transitions between biological outcomes and guides interpretation of the model’s behavior (Liu et al. 2009). Continuation-derived bifurcation maps allow us to chart the global structure of the dynamics and thereby elucidate the conditions under which each clinical scenario (elimination, relapse, or equilibrium, and escape) arises. Numerical continuation plays a key role in confirming the codimension-2 bifurcation points that were identified in the theoretical analysis. When a point such as a Bogdanov–Takens or Bautin (generalized Hopf) is located, continuation can be used to follow the curves emanating from it, for example, the bifurcation curves of saddle-node and Hopf points that meet at a Bogdanov–Takens. This not only corroborates the presence of these organizing centers but also completes the bifurcation portrait by detecting any secondary phenomena (such as homoclinic bifurcations or limit points of cycles) attached to them (Kuznetsov 1998). The analytical and numerical continuation results give a coherent global picture of the model’s dynamics.

**Fig. 5:**
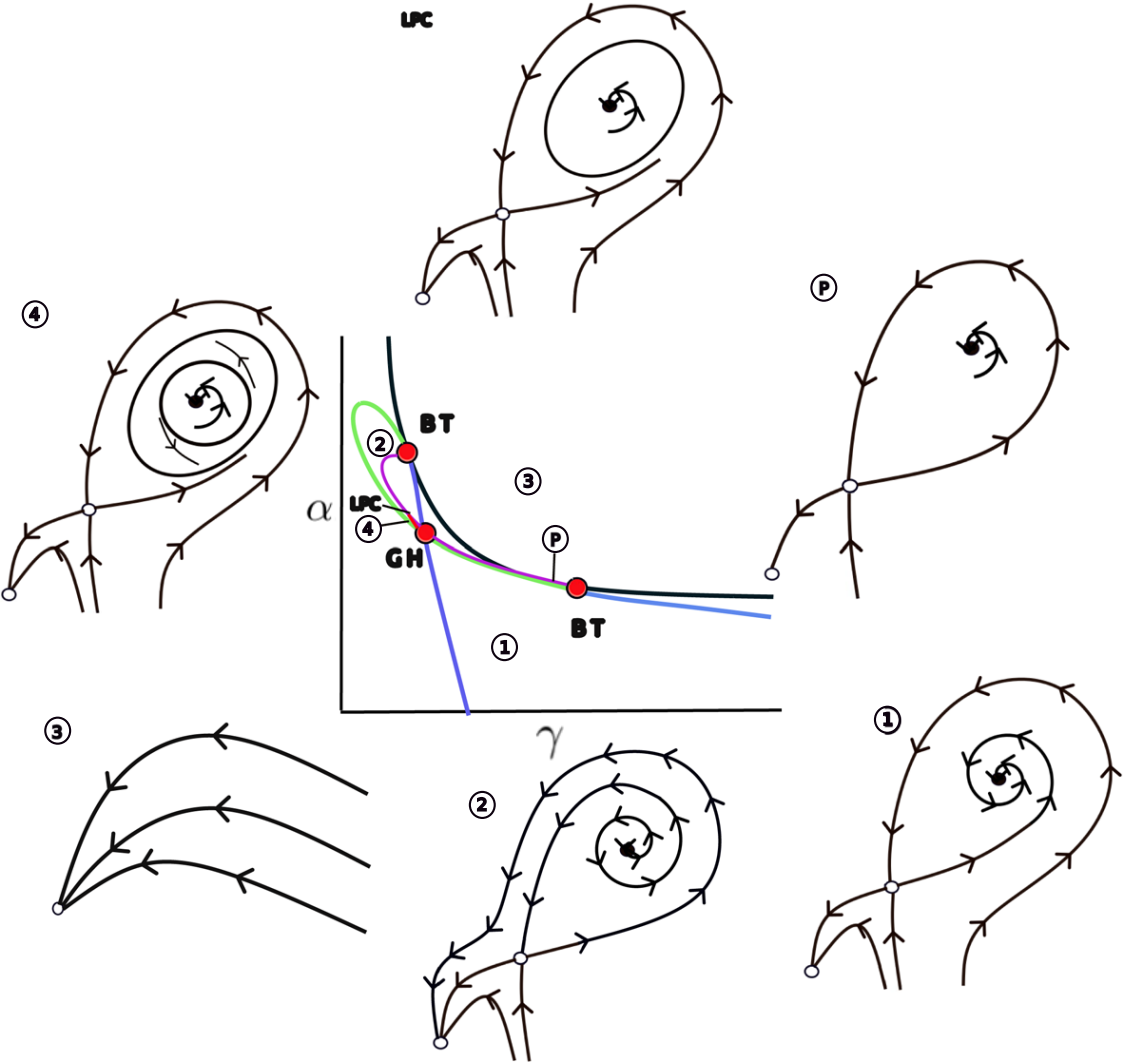
Bifurcation diagram for Model (13) in the $(\gamma , \alpha )$ parameter space, with $\gamma$ as the strong Allee effect threshold and $\alpha$ as the immune elimination rate. The diagram illustrates dynamical regions separated by key bifurcations: green (Hopf), black (saddle-node of critical points). Nested limit cycles emerge from a Generalized Hopf (GH) point on the green curve and collide along the red Limit Point of Cycles (LPC) curve. Bogdanov–Takens (BT) bifurcation points serve as organizers of lower-dimensional bifurcations. As observed, these points delimit the region associated with the set of symmetric saddles (blue) and the set of Hopf bifurcations (green); typically, homoclinic orbit curves (purple) also emerge from them

Figure 5 presents a bifurcation diagram for Model (13), illustrating the impact of key parameters on the system dynamics. Specifically, the diagram shows the system behavior as a function of the strong Allee effect threshold in the cancer cell population ($\gamma$) and the immune system-mediated cancer cell elimination rate ($\alpha$).

As depicted in Figure 5, the parameter space is divided into several distinct regions exhibiting different dynamic behaviors. These regions are described as follows: Region 1 is characterized by the existence of two positive critical points, in addition to the stable critical point at the origin. Similar to the scenario illustrated in Figure 2, one of the positive critical points is stable, while the other is a saddle point. The origin remains a stable critical point throughout this region. The transition to the Region 2 boundary, between Region 1 and Region 2, is marked by a change in stability for the previously stable positive critical point. It becomes unstable through a Hopf bifurcation, represented by the green curve. This mechanism gives rise to limit cycle dynamics. The transition to Region 3 occurs via a saddle-node bifurcation of critical points, represented by the black curve. This bifurcation involves the collision and subsequent disappearance of the two positive critical points. Consequently, in Region 3, the origin remains the critical and stable point. Region 4 is a particularly interesting region located near the Hopf bifurcation curve. Within Region 4, the system exhibits two nested limit cycles. These cycles emerge from a Generalized Hopf (GH) or Bautin bifurcation point (a codimension-2 bifurcation) on the Hopf curve, where the first Lyapunov coefficient vanishes. Originating from the GH point is a curve of Limit Points of Cycles (LPC), shown as the tiny red curve. Along this curve, the two limit cycles collide and annihilate each other, analogous to the saddle-node bifurcation mechanism but occurring with limit cycles. The region delimited by the double limit cycles (often appearing somewhat ‘triangular’ in parameter space) can be very small in practice, potentially leading to numerical precision challenges in locating these cycles, as observed in this case.

The dynamics associated with these double limit cycles are numerically influenced by the presence of homoclinic orbits (the purple curve). These homoclinic orbits emanate from Bogdanov-Takens (BT) bifurcation points and form a curve connecting two such BT points. The interplay between the immune system and cancer cell dynamics in this context is exemplified in Region (P) in Figure 5. A schematic illustration of the LPC mechanism, showing the phase portrait just before the limit cycles disappear, is provided in scheme (LPC). Finally, the blue curves in the diagram represent symmetric saddle bifurcation, which are separated from the green Hopf curve by the BT points.

**Fig. 6:**
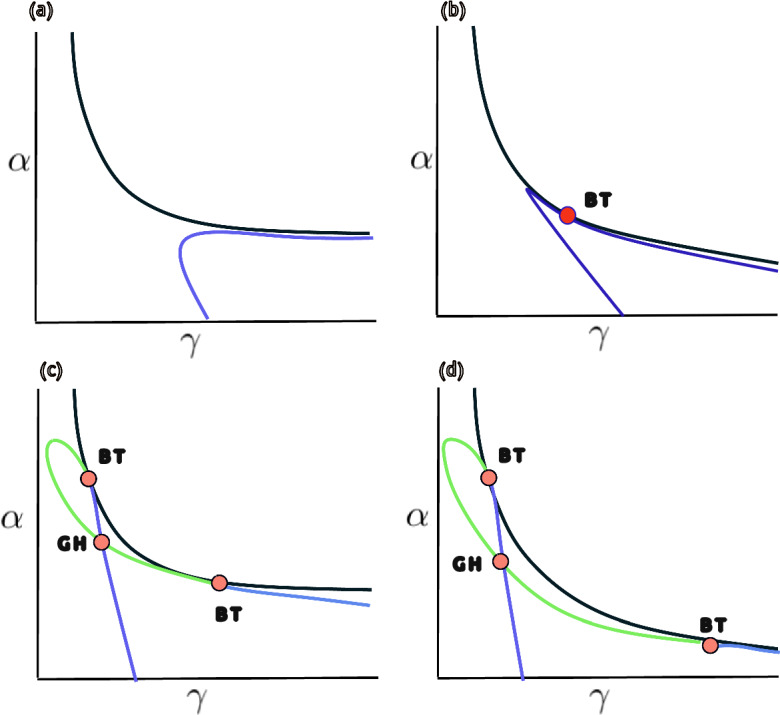
Bifurcation diagram evaluation for Model (13) under variation of parameter *r*. The black curve represents the hypersurface in parameter space associated with saddle-node bifurcations. Blue curves indicate projections of the symmetric saddle-node bifurcations, while the green curve corresponds to the Hopf bifurcation projections. The Hopf bifurcation curve is bounded by two Bogdanov–Takens bifurcations, marked in red with the label **BT**. The generalized Hopf bifurcation, also known as the Bautin bifurcation, is denoted by a red point labeled **GH**. Subfigures correspond to different values of *r*: (a) $r = 0.04$, (b) $r = 0.065$, (c) $r = 0.075$, (d) $r = 0.08$. The remaining parameters are fixed as follows: $b = 0.01$, $\delta = 1$, $\rho = 0$, $\beta = 0.015900262162975$, $\omega = 1$, $\eta = 0$, and $\lambda _1 = 0.2$

**Fig. 7:**
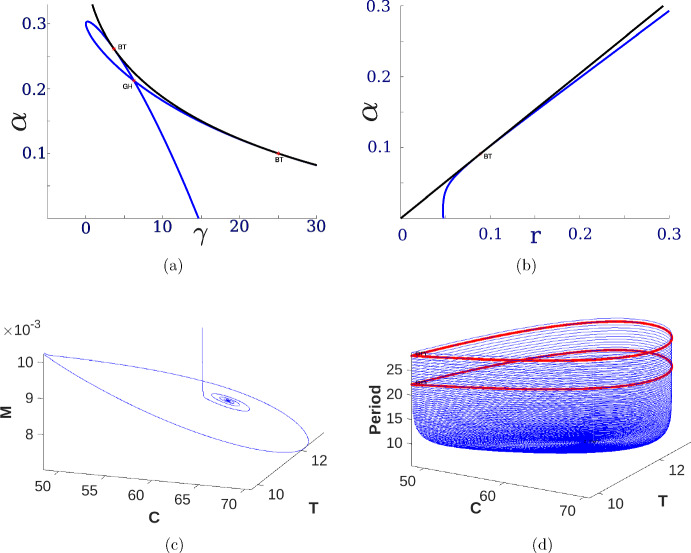
Bifurcation structures and dynamical behaviors of the proposed model (1). (a) Numeric continuation diagram with parameters: $b= 0.01$, $\delta = 1$, $\rho = 0$, $\beta = 0.015900262162975$, $\omega =1$, $\eta =0$, $r=0.08$, and $\lambda =0.2$. The blue curves denote symmetric saddle and Hopf bifurcations, while the black curve denotes saddle-node bifurcations. The BT points are Bogdanov–Takens bifurcations, and the GH point corresponds to the codimension-2 Bautin (generalized Hopf) bifurcation. (b) The blue curve represents symmetric saddles and Hopf bifurcations, and the black curve represents saddle-node bifurcations, in this case the parameters are as follows: $b= 0.01$, $\delta = 1$, $\rho = 0.5$, $\beta = 0.015900262162975$, $\omega =1$, $\eta =0.05$, $\gamma =30$, and $\lambda =0.2$. (c) Dynamics of a numerical limit cycle for the three-variable system, where *C* denotes cancer cells, *M* monocytes, and *T* immune system T cells. (d) Family of limit cycles with varying periods. Red curves denote LPC cycles, showing the balance between variables *C* and *T*

In Figure 6 we present representative bifurcation diagrams in the $(\gamma ,\alpha )$–plane, where $\gamma$ denotes the strong Allee threshold and $\alpha$ the immune–mediated cancer-cell killing rate. In each panel the proliferation rate *r* is varied around its reference value $r_{0}=0.067500003$, while the remaining parameters are fixed at $b=0.01$, $\delta =1$, $\rho =0$, $\beta =0.015900262162975$, $\omega =1$, $\eta =0$, and $\lambda =0.2$. In panel (a), for $r<r_{0}$, no codimension-2 bifurcations appear, indicating a simple structure with only saddle-node and Hopf curves. Panel (b), at $r\approx r_{0}$, reveals the first Bogdanov–Takens (BT) point, marking the onset of higher-order bifurcation phenomena. As *r* increases further in panels (c) and (d), the original BT point gives rise to a Bautin (generalized Hopf) bifurcation together with a second BT point. The BT curves (BT red points) delimit the region in which Hopf bifurcations (green) occur, within which small-amplitude limit cycles coexist with stable equilibria. Although the Bautin point (GH red point) appears to overlap the Hopf curve in the plot, it in fact lies solely on the Hopf curve. This intricate sequence of bifurcations is corroborated by numerical continuation via MATCONT (see as in Figure 7a), and contrasts with the analytically derived loci displayed in Figure 4.

**Fig. 8:**
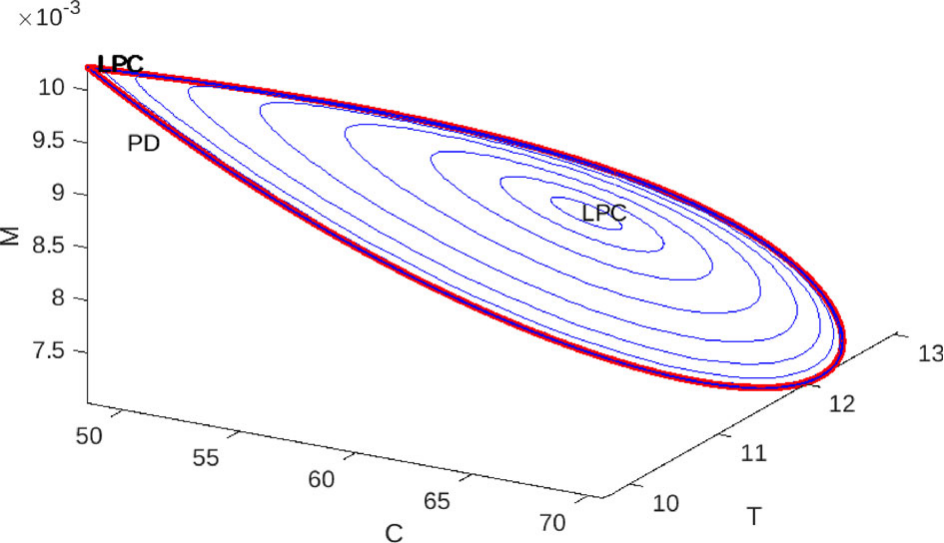
Family of limit cycles for the three-variable system with variables C, T, and M. The parameters for initial point $(C=89.9432,T=17.9902,M=0.00555906)$ are as follows: $b= 0.01$, $\delta = 1$, $\rho = 0.5$, $\beta = 0.015900262162975$, $\omega =1$, $\eta =0$, $r=2$, and $\lambda =0.2$

The three-dimensional dynamical regime of the cancer-immune system interaction is illustrated in Figures 7b, 7c, 7d, and 8, showcasing a family of limit cycles for variables *C* (cancer cells), *M* (monocytes), and *T* (cytotoxic T cells). These closed trajectories reveal sustained oscillatory dynamics driven by the nonlinear coupling between tumor proliferation, innate immune recruitment (*M*), and adaptive immune response (*T*). The varying amplitudes and periods of the cycles reflect distinct regimes of tumor-immune competition, where transient equilibria emerge from the balance between cancer cell growth (*r*) and immune-mediated elimination ($\alpha$). Such oscillations suggest potential tumor dormancy or recurrence mechanisms, as the system alternates between immune dominance and tumor escape phases. The three-dimensional representation further highlights the critical role of monocytes as intermediaries modulating the feedback between *C* and *T*

## Discussion

In this work, we have developed and analyzed a novel mathematical model of cancer–immune interactions that integrates a strong Allee effect in tumor growth with dual pathways of T-cell activation: direct antigenicity-driven stimulation and monocyte-mediated cross-dressing. By coupling ordinary differential equations for cancer cells, effector T lymphocytes, and monocytes, our model reproduces the complex feedback loops and threshold phenomena observed experimentally in murine melanoma studies (Duong et al. 2022; Elewaut et al. 2024). Numerical continuation and bifurcation analysis revealed critical codimension-two organizing centers (Bogdanov–Takens and Bautin points) delineating transitions between tumor clearance, stable coexistence, and escape regimes. These findings extend the biological insights of Elewaut *et al.*, who demonstrated that inflammatory monocytes acquire tumor-derived MHCI–peptide complexes and are essential for intratumoral restimulation of primed CD8^+^ T cells (Magen et al. 2023; Espinosa-Carrasco et al. 2024; Kruse et al. 2023; Elewaut et al. 2024).

In our theoretical framework, the term

$$\begin{aligned} \beta M(C)\;=\;\frac{\beta \,\rho }{\eta + \omega \,C} \end{aligned}$$

quantifies the capacity of monocytes to activate cytotoxic T lymphocytes (CTLs), and its progressive diminution, whether due to tumor-mediated suppression of monocytes (via $\omega C$) or impaired antigen presentation, directly undermines the immune system’s ability to control cancer; similarly, the antigenicity term $\lambda C$ captures T-cell stimulation driven by the tumor’s antigenic visibility, and the combined signal

$$\begin{aligned} C\,\lambda \;+\;\beta M(C)\,T \end{aligned}$$

models the dual activation required in the tumor microenvironment: when both components are high, the cancer-free equilibrium is stable and CTLs eliminate the tumor, whereas if either signal is insufficient, the integrated activation fails to exceed the critical threshold, trapping the system in an immune-evasion regime in which the tumor persists and proliferates unchecked.

The reduced model obtained through the quasi–steady-state approximation (QSSA) constitutes an important conceptual tool to isolate the core mechanisms driving tumor–immune interaction. From a mathematical standpoint, the validity of this reduction relies on the fast–slow structure of the full system: monocyte dynamics evolve on a faster timescale than those of tumor cells and cytotoxic T lymphocytes. Classical geometric singular perturbation theory from Fenichel (1979); Jones (1995); Kuehn (2015) ensures that, when the critical manifold is normally hyperbolic, the positive equilibria of the full system persist under the reduction, and their coordinates differ by $O(\varepsilon )$, where $\varepsilon$ measures the ratio of slow to fast timescales. Under these conditions, the qualitative bifurcation structure, including saddle-node, Hopf, and codimension-two organizing centers, is preserved in the reduced model.

This theoretical expectation is consistent with the numerical results presented in the manuscript. Direct comparisons between the full three-dimensional system (1) and the two-dimensional QSSA reduction (13) show that the positive equilibria and the major bifurcation loci coincide to within numerical precision in the biologically relevant parameter domain considered here. Although codimension-two neighborhoods are—in principle—more sensitive to parameter perturbations, we located these bifurcations in the full model and confirmed their counterparts in the reduced system.

Our results suggest that modulation of monocyte recruitment or cross-dressing efficiency could shift the system across bifurcation boundaries, offering quantitative predictions for combination immunotherapies to sustain antitumor immunity. Moreover, the model highlights the dual role of antigenicity (parameter $\lambda$) and monocyte-mediated activation (parameter $\beta$) in shaping the immune response, and identifies key parameter regimes where small perturbations may precipitate large-scale changes in tumor persistence.

## Conclusion

We have developed a mechanistic ODE model that captures essential immunological processes, including tumor antigenicity, a strong Allee effect, and monocyte-driven cross-dressing, to elucidate the non-linear dynamics of cancer-immune interactions. Analytical and numerical bifurcation analyzes reveal that small changes in monocyte-mediated activation ($\beta$) or the Allee threshold ($\gamma$) can trigger codimension-one and codimension-two bifurcations, leading to qualitatively distinct outcomes: complete tumor eradication, stable tumor–immune coexistence, bistable responses dependent on initial conditions or immune escape. The reduced quasi-steady-state model faithfully preserves these dynamics, allowing for efficient mapping of the parameter space and identification of critical thresholds. In particular, the emergence of Bogdanov–Takens and Bautin points highlights regions where the system is most sensitive to therapeutic perturbations, suggesting that interventions which simultaneously lower the Allee threshold or enhance monocyte cross-dressing may achieve durable tumor control.

This bifurcation-guided modeling approach offers a quantitative blueprint for preclinical testing of immunotherapeutic strategies, enabling the design of combination protocols (e.g., monocyte recruitment enhancers with checkpoint inhibitors) that steer the system across bifurcation boundaries toward favorable attractors. Future work will extend this framework to patient-specific parameter estimation and stochastic modeling to account for intratumoral heterogeneity and adaptive immune variability.

## Figures and Tables

**Fig. 9:**
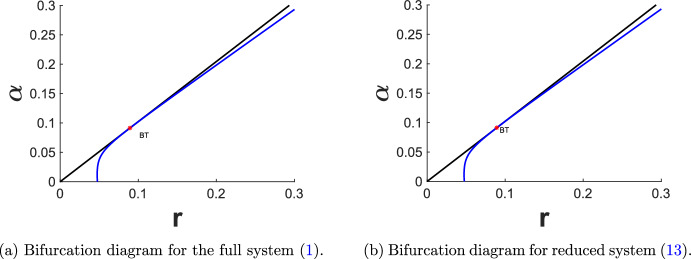
**Numerical Comparison of Bifurcation Diagrams in terms of parameters** *r* **and** $\alpha$. Bifurcation diagram in the parameter space of the full system (1), (a) and the reduced system (13) (b). The curves delineate different bifurcation manifolds: the black curve represents the saddle-node (SN) bifurcations, the blue curve indicates the manifolds corresponding to symmetric saddle and Hopf bifurcations. The fixed parameter values used for continuation are as follows: $b= 0.01$, $\delta = 1$, $\rho = 0.5$, $\beta = 0.015900262162975$, $\omega =1$, $\eta =0.05$, $\gamma =30$, and $\lambda =0.2$

**Fig. 10:**
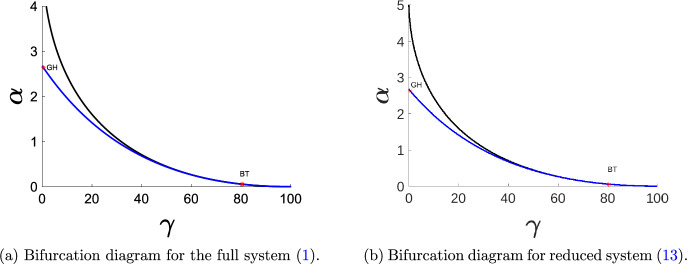
**Numerical Comparison of Bifurcation Diagrams in terms of parameters** $\gamma$ **and** $\alpha$. Bifurcation diagram in the parameter space of the full system (1), (a) and the reduced system (13) (b). The curves delineate different bifurcation manifolds: the black curve represents the saddle-node (SN) bifurcations, the blue curve indicates the manifolds corresponding to symmetric saddle and Hopf bifurcations. The fixed parameter values used for continuation are as follows: $b= 0.01$, $\delta = 1$, $\rho = 0.5$, $\beta = 0.015900262162975$, $\omega =1$, $\eta =0.05$, $r=2$, and $\lambda =0.2$

**Fig. 11:**
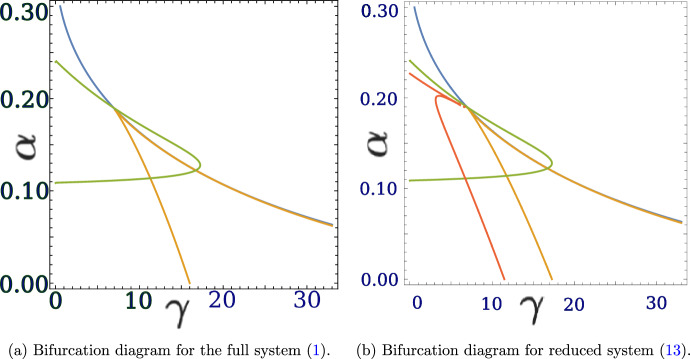
**Analytical Comparison of Bifurcation Diagrams.** Bifurcation diagram in the parameter space of the full system (1), (a) and the reduced system (13) (b). The curves delineate different bifurcation manifolds: the blue curve represents the saddle-node (SN) bifurcations; the yellow curve indicates the manifolds corresponding to symmetric saddle and Hopf bifurcations; the green curve marks the Bogdanov–Takens (BT) bifurcations; and the red curve denotes the Bautin (Generalized Hopf) bifurcations. The fixed parameter values used for continuation are as follows: $b= 0.01$, $\delta = 1$, $\rho = 0$, $\beta = 0.015900262162975$, $\omega =1$, $\eta =0$, $\lambda =0.2$, and $\gamma =0.07$

**Fig. 12:**
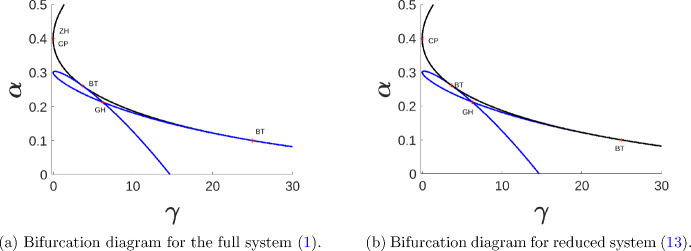
**Numerical Comparison of Bifurcation Diagrams in terms of parameters** $\gamma$ **and** $\alpha$, **with Bautin bifurcation.** Bifurcation diagram in the parameter space of the full system (1), (a) and the reduced system (13) (b). The curves delineate different bifurcation manifolds: the blue curve represents the saddle-node (SN) bifurcations; the yellow curve indicates the manifolds corresponding to symmetric saddle and Hopf bifurcations; the green curve marks the Bogdanov–Takens (BT) bifurcations. The fixed parameter values used for continuation are as follows: $b= 0.01$, $\delta = 1$, $\rho = 0$, $\beta = 0.015900262162975$, $\omega =1$, $\eta =0$, $\lambda =0.2$, and $r=0.08$

**Fig. 13:**
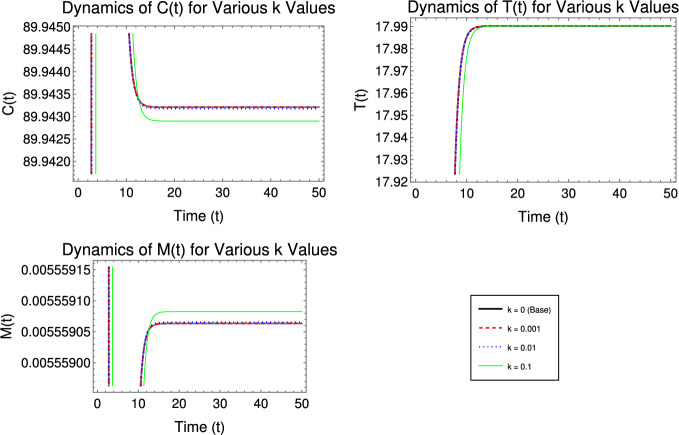
The qualitative structure of the equilibrium curves is preserved for biologically realistic values of the parameter $\kappa$, demonstrating that the term $\kappa MC$ minimally perturbs the system (1)

## Data Availability

No data were used in this study.

## Extended Expressions

This section includes full algebraic expressions.

29 $$\begin{aligned} SN_r= &  \beta ^2 b^4 \gamma ^2 \rho ^2 r^3 (\beta \rho - \gamma \delta \omega )^2 \nonumber \\ &  - 2 \beta b^3 \gamma \rho r^2 (\beta \rho + \gamma \delta \omega )\left( \alpha \beta \gamma \lambda \rho \omega + r (\beta \rho - \gamma \delta \omega )^2 \right) \nonumber \\ &  + b^2 r \left( \alpha ^2 \beta ^2 \gamma ^2 \lambda ^2 \rho ^2 \omega ^2 + r^2 (\beta \rho - \gamma \delta \omega )^2 (\beta ^2 \rho ^2 + 4 \beta \gamma \delta \rho \omega + \gamma ^2 \delta ^2 \omega ^2) \right. \nonumber \\ &  + \left. 4 \alpha \beta \gamma \lambda \rho r \omega (2 \beta ^2 \rho ^2 - \beta \gamma \delta \rho \omega + 2 \gamma ^2 \delta ^2 \omega ^2) \right) \nonumber \\ &  - 2 b r \omega (\beta \rho + \gamma \delta \omega ) \left( 5 \alpha ^2 \beta \gamma \lambda ^2 \rho \omega + \delta r^2 (\beta \rho - \gamma \delta \omega )^2 \right. \nonumber \\ &  + \left. \alpha \lambda r (\beta ^2 \rho ^2 + \beta \gamma \delta \rho \omega + \gamma ^2 \delta ^2 \omega ^2) \right) \nonumber \\ &  + \omega ^2 (\delta r - \alpha \lambda )^2 \left( 4 \alpha \beta \gamma \lambda \rho \omega + r (\beta \rho - \gamma \delta \omega )^2 \right) . \end{aligned}$$

30 $$\begin{aligned} Hopf_r= &  b^4 \beta \gamma ^3 \rho r^4 \omega (2\beta \rho - \gamma \delta \omega ) \nonumber \\ &  + b^3 r^3 \left( -2\beta ^3 \rho ^3 + \beta \gamma ^2 \rho \omega ^2 (2 \alpha \gamma \lambda - \delta ^2 + 2 \gamma ^2 r^2 - 2 \gamma \delta r) \right. \nonumber \\ &  + \left. \beta ^2 \gamma \rho ^2 \omega (3 \delta - 2 \gamma r) + \gamma ^4 \delta r \omega ^3 (\delta - \gamma r) \right) \nonumber \\ &  + b^2 r^2 \omega \left( \beta ^2 \rho ^2 (6 \alpha \lambda - 2 \gamma r^2 + 3 \delta r) \right. \nonumber \\ &  \left. + 2 \beta \gamma \rho r \omega (5 \alpha \gamma \lambda - 2 \delta ^2 - 2 \gamma ^2 r^2 + 3 \gamma \delta r) \right. \nonumber \\ &  + \left. \gamma ^2 \omega ^2 \left( \alpha \lambda (\delta ^2 + 2 \gamma ^2 r^2 - 4 \gamma \delta r) + \delta r (\delta ^2 + \gamma ^2 r^2 - \gamma \delta r) \right) \right) \nonumber \\ &  + b r \omega \left( 2 \beta ^2 \rho ^2 r^3 + \omega ^2 \left( \gamma \delta r^2 (\delta ^2 + \gamma ^2 r^2 - \gamma \delta r) \right. \right. \nonumber \\ &  + \left. \left. \alpha \lambda (\delta ^3 + 4 \gamma ^3 r^3 - 4 \gamma ^2 \delta r^2 - 2 \gamma \delta ^2 r) + \alpha ^2 \gamma \lambda ^2 (4 \gamma r - 3 \delta ) \right) \right. \nonumber \\ &  + \left. \beta \rho \omega (-6 \alpha ^2 \lambda ^2 + 2 \gamma ^2 r^4 - 2 \gamma \delta r^3 - r^2 (\delta ^2 - 10 \alpha \gamma \lambda )) \right) \nonumber \\ &  + \omega ^2 (\delta r - 2 \alpha \lambda ) \left( \beta \rho (-r^3) - \omega (\alpha \lambda + \gamma r^2)(\alpha \lambda + \gamma r^2 - \delta r) \right) . \end{aligned}$$

31 $$\begin{aligned} BT_r= &  b^4 \beta ^3 \rho ^3 \left( 81 \beta ^3 \rho ^3 - 162 \beta ^2 \gamma \delta \omega \rho ^2 + 12 \beta \gamma ^2 (9 \delta ^2 - 4 \alpha \gamma \lambda ) \omega ^2 \rho \right. \nonumber \\ &  \left. + 8 \gamma ^3 \delta (2 \alpha \gamma \lambda - 3 \delta ^2) \omega ^3 \right) r^4 \nonumber \\ &  + b^3 \beta ^2 \rho ^2 \omega \left( -54 \beta ^3 (3 r \delta + 4 \alpha \lambda ) \rho ^3 + 9 \beta ^2 \gamma (33 r \delta ^2 + 10 \alpha \lambda \delta - 4 r \alpha \gamma \lambda ) \omega \rho ^2 \right. \nonumber \\ &  + \left. 4 \beta \gamma ^2 (-45 r \delta ^3 + 22 r \alpha \gamma \lambda \delta - 4 \alpha ^2 \gamma \lambda ^2) \omega ^2 \rho + 12 r \gamma ^3 \delta ^2 (3 \delta ^2 - 2 \alpha \gamma \lambda ) \omega ^3 \right) r^3 \nonumber \\ &  + b^2 \beta \rho \omega ^2 \left( 18 \beta ^3 \left( (6 \delta ^2 - 2 \alpha \gamma \lambda ) r^2 + 5 \alpha \delta \lambda r + 9 \alpha ^2 \lambda ^2 \right) \rho ^3 \right. \nonumber \\ &  + 3 \beta ^2 \left( 6 \gamma \delta (3 \alpha \gamma \lambda - 10 \delta ^2) r^2 + \alpha \gamma \lambda (27 \delta ^2 - 40 \alpha \gamma \lambda ) r + 6 \alpha \delta \lambda (\alpha \gamma \lambda - 5 \delta ^2) \right) \omega \rho ^2 \nonumber \\ &  + \beta \gamma \delta \left( 99 r^2 \gamma \delta ^3 + 6 \alpha (-9 r^2 \gamma ^2 - 8 r \delta \gamma + 7 \delta ^2) \lambda \delta + 28 \alpha ^2 \gamma (2 r \gamma - \delta ) \lambda ^2 \right) \omega ^2 \rho \nonumber \\ &  + \left. 6 r^2 \gamma ^3 \delta ^3 (2 \alpha \gamma \lambda - 3 \delta ^2) \omega ^3 \right) r^2 \nonumber \\ &  + b \omega ^2 \left( -48 r^3 \alpha \beta ^4 \lambda \rho ^4 + 2 r \beta ^3 (-12 r^2 \delta ^3 + 44 r^2 \alpha \gamma \lambda \delta + 3 \alpha ^2 (3 \delta - 20 r \gamma ) \lambda ^2 ) \omega \rho ^3 \right. \nonumber \\ &  + \beta ^2 \left( 36 r^3 \gamma \delta ^4 - 6 r \alpha (9 r^2 \gamma ^2 + 8 r \delta \gamma - 7 \delta ^2) \lambda \delta ^2 + 18 \alpha ^3 \gamma (3 \delta - 2 r \gamma ) \lambda ^3 \right. \nonumber \\ &  + \left. \alpha ^2 (12 r^3 \gamma ^3 + 100 r^2 \delta \gamma ^2 - 17 r \delta ^2 \gamma - 36 \delta ^3) \lambda ^2 \right) \omega ^2 \rho ^2 \nonumber \\ &  + \beta \delta \left( -18 r^3 \gamma ^2 \delta ^4 - 3 \alpha (-6 r^3 \gamma ^3 - 5 r^2 \delta \gamma ^2 + 4 r \delta ^2 \gamma + \delta ^3) \lambda \delta ^2 \right. \nonumber \\ &  - \left. 2 \alpha ^2 \gamma (2 r \gamma - \delta ) (r^2 \gamma ^2 + 6 r \delta \gamma + \delta ^2) \lambda ^2 \right) \omega ^3 \rho \nonumber \\ &  + \left. r^3 \gamma ^3 \delta ^4 (3 \delta ^2 - 2 \alpha \gamma \lambda ) \omega ^4 \right) r \nonumber \\ &  + \alpha \lambda \omega ^3 ( 16 r^3 \beta ^3 (r \delta - \alpha \lambda ) \rho ^3 - \beta ^2 \left( 24 \gamma \delta ^2 r^4 + 28 \alpha \delta (\delta - 2 r \gamma ) \lambda r^2 \right. \nonumber \\ &  + \beta \left( 12 \gamma ^2 \delta ^3 r^4 - 2 \alpha (2 r \gamma - \delta ) \delta (r^2 \gamma ^2 + 6 r \delta \gamma + \delta ^2) \lambda r \right. \nonumber \\ &  + \left. \alpha ^2 (4 r^3 \gamma ^3 + 6 r^2 \delta \gamma ^2 - 9 r \delta ^2 \gamma - 2 \delta ^3) \lambda ^2 \right) \omega ^2 \rho \nonumber \\ &  + \left. r^3 \gamma ^3 \delta ^2 (-2 r \delta ^2 + 2 \alpha \lambda \delta + r \alpha \gamma \lambda ) \omega ^3 \right) \nonumber \\ &  + 18 \alpha ^2 (2 r \gamma - 3 \delta ) \lambda ^2 r + 27 \alpha ^3 \lambda ^3 ) \omega \rho ^2 \end{aligned}$$

32 $$\begin{aligned} \sigma _{Bau}= &  -\beta ^4 \rho ^4 + \beta C^3 \rho r \omega ^3 \Bigg ( \delta \Bigg ( 2 C r \Big ( -18 b^2 C^3 + b \gamma ^2 + 15 b C^2 (b \gamma + 1) \nonumber \\ &  - 4 C (b \gamma + 1)^2 + \gamma \Big ) \nonumber \\ &  + \delta \Big ( C (8 b \gamma - 27 b C + 8) + \gamma \Big ) + \alpha C^2 \lambda \big ( b (\gamma + 6 C) + 1 \big ) \Bigg ) \Bigg ) \nonumber \\ &  + C^5 \delta \omega ^4 \Bigg ( \delta r \Big ( 18 b^2 C^3 r - 15 b C^2 r (b \gamma + 1) + 9 b C \delta + 4 C r (b \gamma + 1)^2 \nonumber \\ &  - (b \gamma + 1)(3 \delta + \gamma r) \Big ) \nonumber \\ &  + \alpha \lambda \big ( \delta - 2 C (b \gamma r + r) \big ) \Bigg ) \nonumber \\ &  + \beta ^2 C^2 \rho ^2 \omega ^2 \Bigg ( 18 b^2 C^4 r^2 - 15 b C^3 r^2 (b \gamma + 1) + C^2 r \Big ( 27 b \delta + 4 r (b \gamma + 1)^2 \Big ) \nonumber \\ &  - C r (b \gamma + 1)(7 \delta + \gamma r) - \alpha C \lambda - \delta (\delta + 2 \gamma r) \Bigg ) \nonumber \\ &  + \beta ^3 C \rho ^3 \omega \Big ( r \big ( C (2 b \gamma - 9 b C + 2) + \gamma \big ) + 2 \delta \Big ) \end{aligned}$$

33 $$\begin{aligned} \text {Hopf}_{C,T,M, 1} &  = b^6 \beta ^2 \rho ^2 \left( 4 \alpha \beta ^4 \lambda \rho ^4 + \omega \right) \left( \beta ^2 \omega \rho ^2 + r \left( \beta ^2 \rho ^2 - 1 \right) \right) r^6 \nonumber \\ &  \quad + b^5 \beta \rho \omega \left( 2 r \alpha \beta ^3 \lambda \rho ^3 \left( -6 r \beta ^3 \rho ^3 + 2 r \beta \rho + \alpha \lambda (1 - \beta \rho (\beta \rho (10 \beta \rho + 1) - 12)) \right) \right. \nonumber \\ &  \quad + 2 \beta ^2 \left( \alpha \lambda \left( \beta ^4 \rho ^4 - 1 \right) - 2 r \right) \omega ^2 \rho ^2 \nonumber \\ &  \quad - \omega \left( 2 \alpha ^2 \beta ^5 \lambda ^2 (12 \beta \rho + 1) \rho ^5 \right. \nonumber \\ &  \quad \left. \left. + r^2 \left( 4 \beta ^2 \rho ^2 - 2 \right) + r \alpha \lambda \left( 10 \beta ^6 \rho ^6 + 2 \beta ^4 \rho ^4 + \beta ^2 \rho ^2 - 2 \right) \right) \right) r^5 \nonumber \\ &  \quad + b^4 \omega \left( -4 r^4 \alpha \beta ^7 \lambda \rho ^7 + r \beta ^3 \rho ^3 \omega \left( -r^3 - \alpha \beta \lambda \rho (\beta \rho - 1) (3 \beta \rho - 1) (4 \beta \rho + 1) r^2 \right. \right. \nonumber \\ &  \quad + \alpha ^2 \lambda ^2 (\beta \rho (\beta \rho (28 \beta \rho + 3) + 8) + 1) r \nonumber \\ &  \quad \left. + 2 \alpha ^3 \lambda ^3 (\beta \rho (\beta \rho (20 \beta \rho + 3) - 30) - 4) \right) \nonumber \\ &  \quad + \beta ^2 \rho ^2 \omega ^3 \left( (6 - \beta \rho ) r^2 + \alpha \lambda \left( 1 - 5 \beta ^4 \rho ^4 \right) r + \alpha ^2 \lambda ^2 \left( 1 - 5 \beta ^3 \rho ^3 (2 \beta \rho + 1) \right) \right) \nonumber \\ &  \quad + \omega ^2 \left( 4 \alpha ^3 \beta ^5 \lambda ^3 (15 \beta \rho + 2) \rho ^5 + r^2 \alpha \lambda \left( -4 \beta ^7 \rho ^7 + 8 \beta ^6 \rho ^6 + \beta ^4 \rho ^4 - 2 \beta ^2 \rho ^2 + 2 \right) \right. \nonumber \\ &  \quad \left. \left. + r^3 \left( -3 \beta ^2 (\beta \rho - 2) \rho ^2 - 1 \right) + r \alpha ^2 \lambda ^2 \left( \beta ^3 \rho ^3 (2 \beta \rho (\beta \rho (14 \beta \rho - 1) + 5) + 5) - 1 \right) \right) \right) r^4 \nonumber \\ &  \quad + b^3 \beta \rho \omega ^2 \left( (8 \alpha \beta ^5 \lambda \rho ^5 + 3 \beta \omega \rho ) r^5 + r^4 \left( 2 \alpha ^2 \beta ^4 \lambda ^2 (2 \beta \rho + 1) \rho ^4 \right. \right. \nonumber \\ &  \quad + \alpha \beta \lambda \rho \omega \left( 2 \beta ^3 \rho ^3 (11 \beta \rho - 3) - 3 \right) \nonumber \\ &  \quad \left. + (9 \beta \rho - 4) \omega ^2 \right) + r^3 \omega \left( \alpha ^2 \beta \rho \lambda ^2 (\beta \rho (\beta \rho (\beta \rho (28 \beta \rho - 5) - 1) + 12) + 1) \right. \nonumber \\ &  \quad \left. + \alpha \lambda \omega \left( 2 \beta ^4 (\beta \rho - 1) \rho ^4 - 5 \beta \rho + 7 \right) + (3 \beta \rho - 4) \omega ^2 \right) \nonumber \\ &  \quad + r^2 \alpha \lambda \omega \left( -\alpha ^2 \beta \rho \lambda ^2 \left( 12 \beta ^3 \rho ^3 + 72 \beta \rho + 13 \right) \right. \nonumber \\ &  \quad + \alpha \lambda \omega (\beta \rho (\beta \rho (\beta \rho (\beta \rho (16 \beta \rho - 7) + 6) + 8) + 3) - 2) \nonumber \\ &  \quad \left. + 2 \omega ^2 \left( \beta ^4 (2 - \beta \rho ) \rho ^4 + 2 \right) \right) \nonumber \\ &  \quad + r \alpha ^2 \lambda ^2 \omega \left( -2 \alpha ^2 \beta \rho \lambda ^2 (20 \beta \rho + 3) \left( \beta ^2 \rho ^2 - 2 \right) \right. \nonumber \\ &  \quad - \alpha \lambda \omega (\beta \rho (\beta \rho (3 \beta \rho (4 \beta \rho - 3) + 19) + 15) + 1) \nonumber \\ &  \quad \left. + \beta \rho \omega ^2 \left( 2 \beta ^2 (5 \beta \rho + 3) \rho ^2 + 1 \right) \right) \nonumber \\ &  \quad + \alpha ^2 \beta ^2 \lambda ^2 \rho ^2 \omega ^2 \left( -4 \alpha ^2 \beta \rho \lambda ^2 (20 \beta \rho + 3) \right. \nonumber \\ &  \left. \left. + \alpha \lambda \omega (5 \beta \rho (4 \beta \rho + 3) + 1) - \beta \rho \omega ^2 \right) \right) r^3 \end{aligned}$$

34 $$\begin{aligned} \text {Hopf}_{C,T,M, 2}= \quad&b^2 \omega ^3 \left( r^4 \alpha \beta ^4 \lambda \rho ^4 \left( \beta \rho (4 \beta \rho - 5) r^2 + \alpha \lambda (4 \beta \rho - 1) r + 2 \alpha ^2 \lambda ^2 (2 \beta \rho + 1) \right) \right. \nonumber \\&\quad + \alpha ^2 \beta ^2 \lambda ^2 \rho ^2 \omega ^4 (\alpha \lambda + \beta \rho (r + 2 \alpha \lambda )) \nonumber \\&\quad + r \beta \rho \omega \left( (\beta \rho - 3) r^5 + \alpha \lambda r^4 \left( \beta ^3 \rho ^3 (4 \beta \rho (\beta \rho - 3) + 1) + 10 \right) \right. \nonumber \\&\quad + \alpha ^2 \lambda ^2 r^3 \left( -4 \beta ^4 \rho ^4 + 3 \beta ^3 \rho ^3 + 13 \beta \rho - 4 \right) \nonumber \\&\quad + \alpha ^3 \lambda ^3 r^2 \left( \beta \rho (\beta \rho (\beta \rho (1 - 12 \beta \rho ) + 1) - 82) - 18 \right) \nonumber \\&\quad + \alpha ^4 \lambda ^4 r \left( \beta \rho (128 - 3 \beta \rho (4 \beta \rho + 1)) + 23 \right) \nonumber \\&\quad \left. + 2 \alpha ^5 \lambda ^5 (\beta \rho (\beta \rho (10 \beta \rho + 1) - 30) - 4) \right) \nonumber \\&\quad + r^2 \omega ^2 \left( (1 - 3 \beta \rho ) r^4 + \alpha \lambda r^3 \left( \beta ^4 (3 \beta \rho - 1) \rho ^4 + 4 \beta \rho - 3 \right) \right. \nonumber \\&\quad + \alpha ^2 \lambda ^2 r^2 \left( 6 \beta ^5 \rho ^5 + 3 \beta ^4 \rho ^4 - \beta ^3 \rho ^3 + \beta \rho + 3 \right) \nonumber \\&\quad + \alpha ^3 \lambda ^3 r (\beta \rho - 1) (\beta \rho + 1) (2 \beta \rho + 1) \nonumber \\&\quad \left. - \alpha ^4 \beta ^2 \lambda ^4 \rho ^2 (5 \beta \rho (4 \beta \rho + 3) + 1) \right) \nonumber \\&\quad + r^2 \omega ^2 \left( r^5 (\beta \rho (\beta \rho - 9) + 1) + 2 \alpha \lambda r^4 \left( 2 \beta ^5 \rho ^5 + 11 \beta \rho - 2 \right) \right. \nonumber \\&\quad + \alpha ^2 \lambda ^2 r^3 (\beta \rho (\beta \rho (\beta \rho (15 \beta \rho - 2) + 9) - 2) + 6) \nonumber \\&\quad - \alpha ^3 \lambda ^3 r^2 (\beta \rho (\beta \rho (\beta \rho (3 \beta \rho (8 \beta \rho - 1) - 4) + 30) + 26) + 4) \nonumber \\&\quad + \alpha ^4 \lambda ^4 r (\beta \rho (15 - 4 \beta \rho (\beta \rho + 1) (8 \beta \rho - 5)) + 1) \nonumber \\&\quad \left. \left. + 4 \alpha ^5 \beta ^3 \lambda ^5 \rho ^3 (15 \beta \rho + 2) \right) \right) r^2 \nonumber \\&\quad + b \omega ^4 \left( -r^4 \alpha \beta ^4 \lambda \rho ^4 (r - 2 \alpha \lambda ) \left( (4 \beta \rho - 1) r^2 + \alpha \lambda r - 2 \alpha ^2 \lambda ^2 \right) \right. \nonumber \\&\quad + \alpha ^2 \beta ^2 \lambda ^2 \rho ^2 \omega ^4 \left( \beta \rho r^2 + \alpha \lambda (r - \alpha \lambda ) \right) \nonumber \\&\quad + \omega ^3 \left( r (r - \alpha \lambda )^4 - r^2 \alpha \beta ^4 \lambda \rho ^4 \left( r^2 + 6 \alpha ^2 \lambda ^2 \right) \right. \nonumber \\&\quad + \alpha ^2 \beta ^3 \lambda ^2 \rho ^3 \left( r^3 - 3 \alpha \lambda r^2 - 10 \alpha ^2 \lambda ^2 r + 10 \alpha ^3 \lambda ^3 \right) \nonumber \\&\quad \left. + \alpha ^3 \beta ^2 \lambda ^3 \rho ^2 (\alpha \lambda - r) (5 \alpha \lambda - 3 r) \right) \nonumber \\&\quad + r \omega ^2 \left( r^6 (3 - 2 \beta \rho ) + \alpha \lambda r^5 \left( 2 \beta ^4 (\beta \rho - 1) \rho ^4 + 4 \beta \rho - 17 \right) \right. \nonumber \\&\quad + \alpha ^2 \lambda ^2 r^4 \left( \beta ^3 (7 \beta \rho - 5) \rho ^3 + 10 \beta \rho + 38 \right) \nonumber \\&\quad + \alpha ^3 \lambda ^3 r^3 \left( \beta \rho (\beta \rho (2 \beta \rho (5 - 3 \beta \rho ) + 1) - 34) - 42 \right) \nonumber \\&\quad + \alpha ^4 \lambda ^4 r^2 \left( \beta \rho (\beta \rho (\beta \rho (16 \beta \rho - 17) - 4) + 32) + 23 \right) \nonumber \\&\quad + \alpha ^5 \lambda ^5 r \left( \beta \rho (\beta \rho (38 \beta \rho + 5) - 10) - 5 \right) \nonumber \\&\quad \left. - 2 \alpha ^6 \beta ^2 \lambda ^6 \rho ^2 (12 \beta \rho + 1) \right) \nonumber \\&\quad + r \omega \left( (r - 2 \alpha \lambda ) (r - \alpha \lambda )^5 - 2 \beta \rho (r - \alpha \lambda )^3 \left( r^3 - 10 \alpha ^2 \lambda ^2 r + 12 \alpha ^3 \lambda ^3 \right) \right. \nonumber \\&\quad - 2 r^5 \alpha \beta ^5 \lambda \rho ^5 + 2 r^2 \alpha \beta ^4 \lambda \rho ^4 \left( r^3 + \alpha \lambda r^2 - \alpha ^2 \lambda ^2 r - 6 \alpha ^3 \lambda ^3 \right) \nonumber \\&\quad \left. - \alpha ^2 \beta ^3 \lambda ^2 \rho ^3 (\alpha \lambda - r) (2 \alpha \lambda - r) \left( 2 r^2 - \alpha \lambda r + 2 \alpha ^2 \lambda ^2 \right) \right) \nonumber \\&\quad \left. + (r - 2 \alpha \lambda ) \omega ^5 (r - 2 \alpha \lambda + \omega ) \left( \alpha \beta ^4 \lambda \rho ^4 r^6 \right. \right. \nonumber \\&\quad + r \omega \left( (r - \alpha \lambda )^5 - 2 r^2 \alpha ^2 \beta ^3 \lambda ^2 \rho ^3 (r - \alpha \lambda ) - r^4 \alpha \beta ^4 \lambda \rho ^4 \right) \nonumber \\&\quad \left. \left. + \alpha ^2 \beta ^2 \lambda ^2 \rho ^2 \omega ^2 (r - \alpha \lambda ) \left( \beta \rho r^2 + \alpha \lambda (r - \alpha \lambda ) \right) \right) \right) r \nonumber \\&\quad + (r - 2 \alpha \lambda )\omega ^5 (r - 2 \alpha \lambda + \omega )\nonumber \\&\quad \left( \alpha \beta ^4\lambda \rho ^4 r^6 + \left( (r - \alpha \lambda )^5 - 2 r^2\alpha ^2\beta ^3\lambda ^2 \rho ^3 (r - \alpha \lambda ) - r^4\alpha \beta ^4\lambda \rho ^4 \right) \omega r \right. \nonumber \\&\quad \left. + \alpha ^2\beta ^2\lambda ^2 \rho ^2 \omega ^2 (r - \alpha \lambda )\left( \beta \rho r^2 + \alpha \lambda (r - \alpha \lambda ) \right) \right) \end{aligned}$$

35 $$\begin{aligned} \text {BT}_{C,T,M, 1}&= r \beta ^2 \gamma \rho ^2 \omega ^2 \left[ b^4 r^4 \beta ^4 \rho ^4 \left( 3 (3 \beta \rho - 2 \gamma \delta \omega )^3 + 2 \alpha \gamma \lambda \left( 27 \beta ^2 \rho ^2 + 8 \gamma ^3 \delta \omega ^3 \right. \right. \right. \nonumber \\&\quad \left. \left. - 6 \beta \gamma \rho \omega (3 \delta + 4 \gamma \omega ) \right) \right) \nonumber \\&\quad + \alpha \lambda \omega ^3 \left( -\alpha ^2 \beta ^2 \lambda ^2 \rho ^2 \omega (3 \alpha \lambda (4 \delta ^2 + 9 \beta \rho ) + 2 \delta ^3 \omega ) \right. \nonumber \\&\quad + r \alpha \beta \lambda \rho \left( \alpha ^2 \lambda ^2 (\alpha \gamma \lambda + 2 \beta \rho ) (4 \delta ^2 + 9 \beta \rho ) \right. \nonumber \\&\quad + 2 \alpha \delta \lambda \omega (-\alpha \gamma \lambda (5 \delta ^2 + 12 \beta \rho ) + \beta \rho (10 \delta ^2 + 27 \beta \rho )) \nonumber \\&\quad \left. + \delta ^2 \omega ^2 (2 \beta \delta ^2 \rho - \alpha \gamma \lambda (4 \delta ^2 + 9 \beta \rho )) \right) \nonumber \\&\quad + 2 r^2 \alpha \lambda \omega \left( -\alpha \beta \delta \lambda \rho (\alpha \gamma \lambda + 2 \beta \rho ) (4 \delta ^2 + 11 \beta \rho ) \right. \nonumber \\&\quad - \beta \rho \omega \left( 2 \alpha ^2 \gamma ^2 \lambda ^2 (\delta ^2 + 3 \beta \rho ) + 2 \beta \delta ^2 \rho (2 \delta ^2 + 7 \beta \rho ) \right. \nonumber \\&\left. \quad + \alpha \gamma \lambda (-9 \delta ^4 - 20 \beta \delta ^2 \rho + 18 \beta ^2 \rho ^2) \right) \nonumber \\&\quad \left. + \gamma \delta \omega ^2 \left( 2 \beta \delta ^2 \rho (\delta ^2 + 2 \beta \rho ) - \alpha \gamma \lambda (\delta ^4 + 3 \beta \delta ^2 \rho - 3 \beta ^2 \rho ^2) \right) \right) \nonumber \\&\quad + r^3 \alpha \lambda \left( 4 \beta \rho (\alpha \gamma \lambda + 2 \beta \rho ) (\delta ^4 + 3 \beta \delta ^2 \rho - 2 \beta ^2 \rho ^2) \right. \nonumber \\&\quad + 2 \beta \gamma \delta \rho \omega \left( -4 \delta ^4 + \delta ^2 (\alpha \gamma \lambda - 10 \beta \rho ) + 7 \beta \rho (\alpha \gamma \lambda + 4 \beta \rho ) \right) \nonumber \\&\quad + \gamma ^2 \omega ^2 \left( 2 \delta ^6 + 4 \alpha \beta ^2 \gamma \lambda \rho ^2 + \delta ^4 (-\alpha \gamma \lambda + 4 \beta \rho ) - 2 \beta \delta ^2 \rho (\alpha \gamma \lambda + 11 \beta \rho ) \right) \nonumber \\&\quad \left. + 2 \beta \gamma ^3 \delta ^3 \rho \omega ^3 \right) \nonumber \\&\quad \left. + r^4 \beta \delta \rho \left( 2 (2 \beta \rho - \gamma \delta \omega )^3 + \alpha \gamma \lambda \left( 8 \beta ^2 \rho ^2 + \gamma ^3 \delta \omega ^3 - 4 \beta \gamma \rho \omega (\delta + \gamma \omega ) \right) \right) \right] \nonumber \\ \end{aligned}$$

36 $$\begin{aligned}&\text {BT}_{C,T,M, 2}= b^2 r^2 \beta \rho \omega \left( \alpha \beta \lambda \rho \omega \left( 9 \alpha \beta \lambda \rho \left( 3 \delta ^4 + 12 \beta \delta ^2 \rho + 2 \beta \rho (\alpha \gamma \lambda + 9 \beta \rho ) \right) \right. \right. \nonumber \\&\quad + 2 \delta \omega \left( 3 \alpha \gamma \lambda (\delta ^2 + \beta \rho ) (2 \delta ^2 + 3 \beta \rho ) - 9 \beta \delta ^2 \rho (\delta ^2 + 5 \beta \rho ) - 2 \alpha ^2 \gamma ^2 \lambda ^2 (4 \delta ^2 + 11 \beta \rho ) \right) \nonumber \\&\quad \left. + 2 \gamma \delta ^2 \omega ^2 (3 \delta ^2 - 2 \alpha \gamma \lambda ) (2 \delta ^2 + 7 \beta \rho ) \right) \nonumber \\&\quad + r \alpha \lambda \left( -9 \alpha \beta \lambda \rho (\alpha \gamma \lambda + 2 \beta \rho ) (\delta ^4 + 4 \beta \delta ^2 \rho + 5 \beta ^2 \rho ^2) \right. \nonumber \\&\quad + 6 \beta \delta \rho \omega \left( -\alpha ^2 \gamma ^2 \lambda ^2 (\delta ^2 + 4 \beta \rho ) + 3 \beta \rho (\delta ^2 + \beta \rho ) (2 \delta ^2 + 5 \beta \rho ) \right. \nonumber \\&\quad \left. + 2 \alpha \gamma \lambda (3 \delta ^4 + 10 \beta \delta ^2 \rho + 4 \beta ^2 \rho ^2) \right) \nonumber \\&\quad + \gamma \omega ^2 \left( 4 \alpha ^2 \gamma ^2 \lambda ^2 (\delta ^4 + 3 \beta \delta ^2 \rho - 2 \beta ^2 \rho ^2) + 3 \beta \delta ^2 \rho (-14 \delta ^4 - 42 \beta \delta ^2 \rho + 27 \beta ^2 \rho ^2) \right. \nonumber \\&\quad \left. - 6 \alpha \gamma \lambda (\delta ^6 - 10 \beta ^2 \delta ^2 \rho ^2 + 20 \beta ^3 \rho ^3) \right) \nonumber \\&\quad \left. + 2 \gamma ^2 \delta \omega ^3 \left( 6 \delta ^6 + 28 \alpha \beta ^2 \gamma \lambda \rho ^2 - 2 \beta \delta ^2 \rho (5 \alpha \gamma \lambda + 12 \beta \rho ) + \delta ^4 (-4 \alpha \gamma \lambda + 15 \beta \rho ) \right) \right) \nonumber \\&\quad + r^2 \beta \rho \left( 9 \delta ^2 \omega (3 \beta \rho - 2 \gamma \delta \omega ) (-2 \beta \rho + \gamma \delta \omega )^2 \right. \nonumber \\&\quad \left. + \alpha ^2 \gamma \lambda ^2 \left( -4 \gamma ^3 \delta ^2 \omega ^3 + 9 \beta \gamma \delta \rho \omega (\delta + 2 \gamma \omega ) \right. \right. \nonumber \\&\quad \left. - 6 \beta ^2 \rho ^2 (3 \delta + 2 \gamma \omega ) \right) \nonumber \\&\quad + 6 \alpha \lambda \left( -6 \beta ^3 \rho ^3 (\delta + \gamma \omega ) + 9 \beta ^2 \gamma \delta \rho ^2 \omega (2 \delta + \gamma \omega ) + \gamma ^3 \delta ^3 \omega ^3 (\delta + 2 \gamma \omega )\right. \nonumber \\&\quad \left. \left. \left. - \beta \gamma ^2 \delta ^2 \rho \omega ^2 (10 \delta + 9 \gamma \omega ) \right) \right) \right] \nonumber \\&\quad + b^3 r^3 \beta ^2 \rho ^2 \left[ -\alpha \lambda \omega \left( 3 \left( 9 \beta ^2 \rho ^2 (\delta ^4 + 4 \beta \delta ^2 \rho + 8 \beta ^2 \rho ^2) \right. \right. \right. \nonumber \\&\quad - 6 \beta \gamma \delta \rho (\delta ^2 + \beta \rho ) (2 \delta ^2 + 5 \beta \rho ) \omega \nonumber \\&\quad \left. + 4 \gamma ^2 \delta ^4 (\delta ^2 + 3 \beta \rho ) \omega ^2 \right) \nonumber \\&\quad + 2 \alpha \gamma \lambda \left( 9 \beta \rho (\delta ^4 + 4 \beta \delta ^2 \rho + 5 \beta ^2 \rho ^2) + 6 \beta \gamma \delta \rho (\delta ^2 + 4 \beta \rho ) \omega \right. \nonumber \\&\quad \left. \left. - 4 \gamma ^2 (\delta ^4 + 3 \beta \delta ^2 \rho - 2 \beta ^2 \rho ^2) \omega ^2 \right) \right) \nonumber \\&\quad + r \beta \rho \left( 9 \delta \omega (3 \beta \rho - 2 \gamma \delta \omega )^2 (-2 \beta \rho + \gamma \delta \omega ) + \alpha ^2 \gamma \lambda ^2\right. \nonumber \\&\qquad \left( 27 \beta ^2 \rho ^2 + 8 \gamma ^3 \delta \omega ^3 - 6 \beta \gamma \rho \omega (3 \delta + 4 \gamma \omega ) \right) \nonumber \\&\quad + 2 \alpha \lambda \left( 27 \beta ^3 \rho ^3 - 18 \beta ^2 \gamma \rho ^2 \omega (5 \delta + \gamma \omega ) \right. \nonumber \\&\quad \left. \left. \left. - 6 \gamma ^3 \delta ^2 \omega ^3 (\delta + 2 \gamma \omega ) + 2 \beta \gamma ^2 \delta \rho \omega ^2 (27 \delta + 22 \gamma \omega ) \right) \right) \right] \nonumber \\&\quad + b r \omega ^2 \left[ r^2 \alpha \lambda \left( -6 \alpha \beta ^2 \delta \lambda \rho ^2 (\alpha \gamma \lambda + 2 \beta \rho ) (\delta ^2 + 4 \beta \rho ) \right. \right. \nonumber \\&\quad - \beta \rho \omega \left( 12 \beta \delta ^4 \rho (\delta ^2 + 3 \beta \rho ) + \alpha ^2 \gamma ^2 \lambda ^2 (-2 \delta ^4 - 3 \beta \delta ^2 \rho + 40 \beta ^2 \rho ^2) \right. \nonumber \\&\quad \left. + 6 \alpha \gamma \lambda (\delta ^6 - 10 \beta ^2 \delta ^2 \rho ^2 + 20 \beta ^3 \rho ^3) \right) \nonumber \\&\quad + 2 \beta \gamma \delta \rho \omega ^2 \left( 6 \delta ^6 - 5 \delta ^4 (\alpha \gamma \lambda - 3 \beta \rho ) + \alpha \beta \gamma \lambda \rho (7 \alpha \gamma \lambda + 50 \beta \rho ) \right. \nonumber \\&\quad \left. + \delta ^2 (\alpha ^2 \gamma ^2 \lambda ^2 - 17 \alpha \beta \gamma \lambda \rho - 24 \beta ^2 \rho ^2) \right) \nonumber \\&\quad \left. + \gamma ^2 \delta ^2 \omega ^3 \left( -3 \delta ^6 - 22 \alpha \beta ^2 \gamma \lambda \rho ^2 + 2 \delta ^4 (\alpha \gamma \lambda - 3 \beta \rho ) + \beta \delta ^2 \rho (4 \alpha \gamma \lambda + 15 \beta \rho ) \right) \right) \nonumber \\&\quad + \alpha \beta ^2 \lambda \rho ^2 \omega \left( 2 \alpha ^3 \gamma \lambda ^3 (4 \delta ^2 + 9 \beta \rho ) - 3 \delta ^6 \omega ^2 + 2 \alpha \delta ^3 \lambda \omega \left( -9 (\delta ^2 + 2 \beta \rho ) + \gamma \delta \omega \right) \right. \nonumber \\&\quad \left. + 2 \alpha ^2 \delta \lambda ^2 \omega \left( 6 \beta \delta \rho + 10 \gamma \delta ^2 \omega + 27 \beta \gamma \rho \omega \right) \right) \nonumber \\&\quad + r^3 \beta \rho \left( 3 \delta ^3 \omega (-2 \beta \rho + \gamma \delta \omega )^3 - 2 \alpha ^2 \beta \gamma \lambda ^2 \rho \left( 12 \beta ^2 \rho ^2 + \gamma ^2 \delta \omega ^2 (\delta + 2 \gamma \omega ) \right. \right. \nonumber \\&\quad \left. - 3 \beta \gamma \rho \omega (3 \delta + 2 \gamma \omega ) \right) \nonumber \\&\quad + 2 \alpha \lambda \left( -24 \beta ^4 \rho ^4 + 44 \beta ^3 \gamma \delta \rho ^3 \omega - \gamma ^4 \delta ^4 \omega ^4 + 3 \beta \gamma ^2 \delta ^3 \rho \omega ^2 (\delta + 3 \gamma \omega ) \right. \nonumber \\&\quad \left. \left. - 3 \beta ^2 \gamma \delta ^2 \rho ^2 \omega (2 \delta + 9 \gamma \omega ) \right) \right) \nonumber \\&\quad + r \alpha \beta \lambda \rho \left( 6 \delta ^4 \omega ^2 \left( \beta \rho (2 \delta ^2 + 7 \beta \rho ) - \gamma \delta (\delta ^2 + 2 \beta \rho ) \omega \right) \right. \nonumber \\&\quad + \alpha \delta \lambda \omega \left( 6 \beta \rho (\delta ^2 + \beta \rho ) (2 \delta ^2 + 3 \beta \rho ) - \gamma \delta \omega (15 \delta ^4 + 34 \beta \delta ^2 \rho + 17 \beta ^2 \rho ^2) \right. \nonumber \\&\quad \left. + 4 \gamma ^2 \delta ^2 \omega ^2 (\delta ^2 + 2 \beta \rho ) \right) \nonumber \\&\quad + 2 \alpha ^2 \lambda ^2 \left( 9 \beta ^3 \rho ^3 + 3 \gamma \delta ^4 \omega (\delta + 3 \gamma \omega ) - 2 \beta ^2 \gamma \rho ^2 \omega (25 \delta + 9 \gamma \omega ) \right. \nonumber \\&\quad \left. + \beta \gamma \delta ^2 \rho \omega (-7 \delta + 20 \gamma \omega ) \right) \nonumber \\&\quad \left. \left. + \alpha ^3 \gamma \lambda ^3 \left( -8 \gamma \delta ^3 \omega + \beta \rho (9 \beta \rho - 22 \gamma \delta \omega ) \right) \right) \right] \end{aligned}$$

## Reduction Model Clarification and Sensibility

See Figures 9, 10, 11, 12 and 13.

### Justification for Equilibrium Preservation

The **Quasi-Steady-State Approximation (QSSA)** is employed to reduce the full three-dimensional system (1) to a simplified two-dimensional system (13). The validity of this reduction hinges on the preservation of the essential steady-state structure within the biologically relevant parameter space.

For the chosen set of biologically plausible parameters, the QSSA successfully preserves the number and location of the positive, non-trivial equilibrium points. The parameter values used for the calculation are:

$$\begin{aligned} {\textbf{P}}= &  \{b = 0.01, \gamma = 30, \delta = 1, \omega = 1, \beta = 0.0159002, \rho = 0.5, \alpha = 1, \\ &  \lambda = 0.2, r = 2, \eta = 0.5\} \end{aligned}$$

The steady-state solutions for the full and reduced systems are compared in Table 1.

**Table 1:** Comparison of Equilibrium Points for Full and Reduced Systems

Equilibrium Type	Full Model (1)	Reduced Model (13)
Trivial (Boundary)	(0.0, 0.0, 1.0)	(0.0, 0.0)
Non-Biologically Plausible	$(-0.492037, -61.2841, 62.7911)$	$(-0.492037, -61.2841)$
Positive (Non-Trivial 1)	$\mathbf {(35.5067, 7.10291, 0.0138863)}$	$\mathbf {(35.5067, 7.10291)}$
Positive (Non-Trivial 2)	$\mathbf {(84.4933, 16.9002, 0.00588282)}$	$\mathbf {(84.4933, 16.9002)}$

The numerical evidence, Table 1, confirms that the coordinates of the critical points, particularly the two positive and biologically relevant equilibria, are identical between the full system (1) and the reduced system (13) up to the calculated precision.

## Footnotes

1 The explicit expression for ${\mathcal {S}}_r$ is provided in equation (29) in Appendix A.

2 The hypersurface ${Hopf}_r$ is in the Appendix A as (30)

3 As with the expressions $SN_r$ and $Hopf_r$, the full expression for $BT_r$ is provided in Appendix A as (31).
